# Genetic Diversity and Population Structure of Potato Germplasm in RDA-Genebank: Utilization for Breeding and Conservation

**DOI:** 10.3390/plants10040752

**Published:** 2021-04-12

**Authors:** Kyung-Jun Lee, Raveendar Sebastin, Gyu-Taek Cho, Munsup Yoon, Gi-An Lee, Do-Yoon Hyun

**Affiliations:** 1 National Agrobiodiversity Center, National Institute of Agricultural Sciences (NAS), RDA, Jeonju 54874, Jeol-labuk-do, Korea; kiriice@nate.com (K.-J.L.); raveendars@gmail.com (R.S.); gtcho@korea.kr (G.-T.C.); msyoon63@korea.kr (M.Y.); gkntl1@korea.kr (G.-A.L.); 2 Honam National Institute of Biological Resources, 99, Gohadoan-gil, Mokpo-si 58762, Jeollanam-do, Korea

**Keywords:** potato, genetic diversity, plant germplasm, SSR

## Abstract

Potato (*Solanum tuberosum* L.) is an important staple food and economic crop in many countries. It is of critical importance to understand the genetic diversity and population structure for effective collection, conservation, and utilization of potato germplasm. Thus, the objective of the present study was to investigate the genetic diversity and population structure of potato germplasm conserved in the National Agrobiodiversity Center (NAC) of South Korea to provide basic data for future preservation and breeding of potato genetic resources. A total of 24 simple sequence repeat (SSR) markers were used to assess the genetic diversity and population structure of 482 potato accessions. A total of 257 alleles were detected, with an average of 10.71 alleles per locus. Analysis of molecular variance showed that 97% of allelic diversity was attributed to individual accessions within the population, while only 3% was distributed among populations. Results of genetic structure analysis based on STRUCTURE and discriminant analysis of principal components revealed that 482 potato accessions could be divided into two main subpopulations. Accessions of subpopulation 1 mainly belonged to cultivars and breeding lines. Accessions of subpopulations 2 basically corresponded to wild relatives of potatoes. Results of this study provide useful information for potato improvement and conservation programs, although further studies are needed for a more accurate evaluation of genetic diversity and phenotypic traits of potatoes.

## 1. Introduction

Potato (*Solanum tuberosum*) is one of the most important tuber crops. It can be used as raw material for starch and alcohol production [1]. Potato is the world’s fourth most important food crop after maize, wheat, and rice [2]. According to the Food and Agricultural Organization of the United Nations, global potato production was 462 million tons in 2017. Its production has increased steadily over the years. In 2019, South Korea was the 55th potato-producing country in the world, producing 630,140 tons [2]. 

The cultivated potato was domesticated 8000–10,000 years ago from diploid wild species (2x = 2n = 24) native to the Andes of Southern Peru [3]. Its migration from the Andes to coastal Chile caused its adaptation to long-day conditions. This improved potato germplasm later contributed greatly to the development of commercial cultivars worldwide [4]. Since its domestication, it has been widely adopted into the human diet and has become the most important non-cereal staple food across the globe. Potato is an important food crop, serving as a major source of calories and contributing to food security in Asia and South America [5]. In the early 18th century, cultivated potatoes in Korea came from China. Since the 20th century, various potato varieties have been introduced from Japan, the United States, and Europe. Currently, various potato varieties have been developed according to their applications, with foreign-introduced potato varieties being widely cultivated [6].

Wild relatives are important to agricultural crops because they have been extremely valuable in adapting crop varieties to changing disease pressures, farming practices, market demands, and climatic conditions [7]. There are some 200 species in *Solanum* section *Petota*, but all advanced cultivars of potato are collectively classified under the single species, *S. tuberosum* L. [8,9]. Species of set. *Petota* have a base chromosome number of x = 12. There are diploids (2n = 2x = 24), triploids (2n = 36), tetraploids (2n = 48), pentaploids (2n = 60), and hexaploids (2n−72) [10]. Although polyploidy is common in set. Petota, the modes of origin of polyploidy in set. *Petota* remain largely unresolved [11]. Potato wild relatives could be used for various purposes in potato breeding program, such as disease resistance and environmental tolerance [12,13,14]. The efficient use of wild relatives now requires extensive knowledge of their allelic variation and genomic structure, including the screening for desirable traits [15].

Plant genetic resources are among the most essential natural resources of the world. Major advances have been made in conserving them [16]. Gene banks are concerned with the maintenance of genetic variations of crop genetic resources because plant genetic resource conservation merits far greater attention than it is now receiving [16,17]. To establish effective and efficient conservation practices for plant genetic resources, understanding the genetic diversity between and within populations is important [18].

Molecular techniques have been widely adopted as powerful tools for germplasm characterization, cultivar identification, phylogenetic studies, and diversity analysis of many crop plants [19]. In the study of potato, random amplified polymorphic DNA (RAPD) [20,21,22], inter-retrotransposon amplified polymorphism (IRAP) [23], inter-simple sequence repeats (ISSRs) [24], amplified fragment length polymorphism (AFLP) [25,26,27], and simple sequence repeats (SSRs) [19,25,26,27,28,29] have been used for the estimation of genetic diversity and genetic relationships. Among DNA markers, SSRs have been used successfully in polyploid species such as *Brassica napus* [30,31], *Arachis* [32], ginseng [33], tea [34], sweet potato [19], and potato species [28,35]. SSR markers have been preferred due to their co-dominance, random genome distribution, high level of polymorphism, simplicity of use, high clarity and reproducibility, low operational cost, hyper-variability, amenability to automation, ease of multiplexing, and use with low quality DNA [19,26,28,35]. SSR markers have been widely used in the determination of genetic diversity, germplasm fingerprinting, heterosis analysis, tracing germplasm migrations, gene flow, genetic linkage mapping, and phylogenetic studies.

The conservation of cultivated potato species and their wild relatives in gene banks provides long-term availability of crop genetic diversity [36]. Characterizing these collections is essential to identify alleles/genes associated with traits of interest for plant breeding, such as resistance to pathogens and insect pests, tolerance of abiotic stresses (e.g., salinity and frost), and tuber quality [37,38]. In Korea, 1666 potato genetic resources (31 *Solanum* species) are maintained in the National Agrobiodiversity Center (NAC) at Rural Development Administration (RDA). However, these preserved potato accessions in the NAC focus on the maintenance of genetic resources without much analysis of their genetic diversity. In the present study, 24 SSR primer pairs were used to analyze 482 potato accessions collected in the NAC with the following objectives: (1) To evaluate the population structure of potato accessions; and (2) to estimate genetic differentiation and variation source among inferred populations. Results of this study provide important information that can help us deeply understand the genetic diversity, population structure, and differentiation of potato varieties to guide effective collection, conservation, and application of potato genetic resources in Korea.

## 2. Results

A total of 257 alleles were detected in 24 SSR loci among 482 potato accessions (Table 1). The number of observed alleles (Na) ranged from 4 (STM5121) to 20 (STM0019a,b), with an average number of 10.7. The number of genotypes (Ng) ranged from 6 (STM1053) to 60 (STM0019a,b) with an average number of 30.3. The Shannon–Wiener index (H) ranged from 0.39 (STM1053) to 2.00 (STM0019a,b) with an average of 1.44. Nei’s genetic diversity (GD) was calculated to be from 0.18 (STM1053) to 0.83 (STM0019a,b) with an average of 0.68. Evenness value ranged from 0.46 (STM1053) to 0.91 (STM 0032) with an average of 0.75.

Diversity indices among seven origins were calculated. Results are shown in Table 2. Na ranged from 5.46 (BGR) to 9.88 (USA), and Ng ranged from 9.00 (BGR) to 21.88 (USA). H were calculated to be 1.28 (CHN) to 1.51 (USA), GD and Evenness ranged from 0.66 (CHN, KOR, and NLD) to 0.70 (USA) and from 0.74 (USA) to 0.82 (CHN), respectively.

Analysis of molecular variance (AMOVA) on genetic differentiation among and within the population of potato accessions was conducted according to their seven origins. Results are shown in Table 3. Findings from AMOVA revealed that 97% of total genetic variations were contributed by differences within populations. This percentage was notably and significantly higher than that among populations (only 3% of total genetic variation was due to differences among populations). PhiPT and gene flow (Nm, Nm > 1.0, which shows little differentiation among populations) for 482 potato accessions were 0.031 (*p* < 0.0001) and 15.828, respectively. Pairwise population PhiPT values for seven origins ranged from 0.008 (CHN-JPN) to 0.095 (KOR-PER) (Table 4). Pairwise population estimates of gene flow for seven origins ranged from 4.775 (KOR-PER) to 63.589 (CHN-JPN).

**Figure 1:**
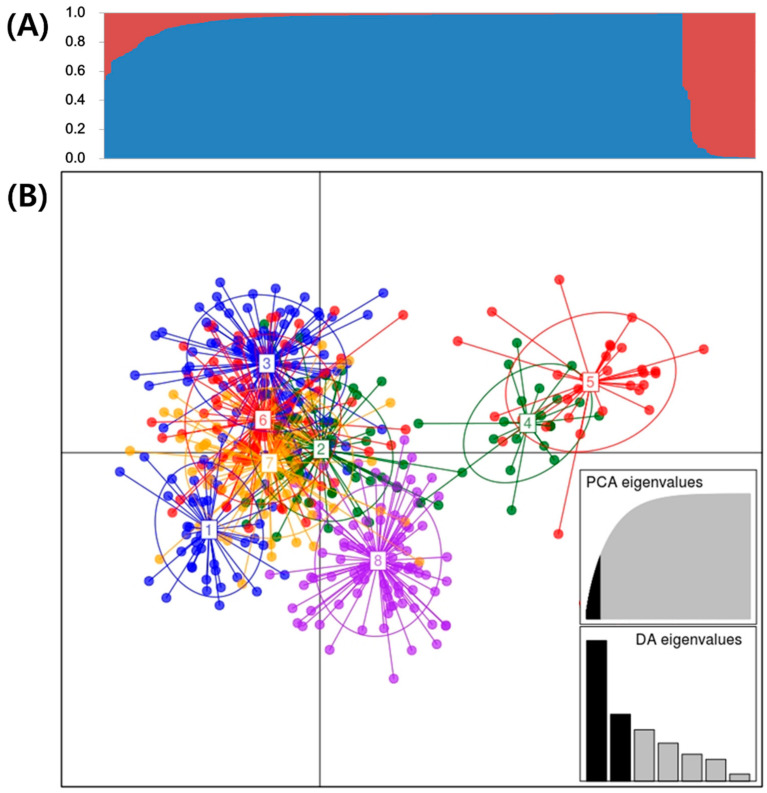
(**A**) Population structure analysis of 482 potato accessions inferred using STRUCTURE software based on 24 SSR markers for delta K = 2. (**B**) Discriminant analysis of principal components (DAPC) for 482 potato accessions. Axes represent the first two Linear Discriminants (LD). Each circle represents a cluster. Each dot represents an individual. Numbers represent different clusters identified by DAPC analysis.

**Figure 2:**
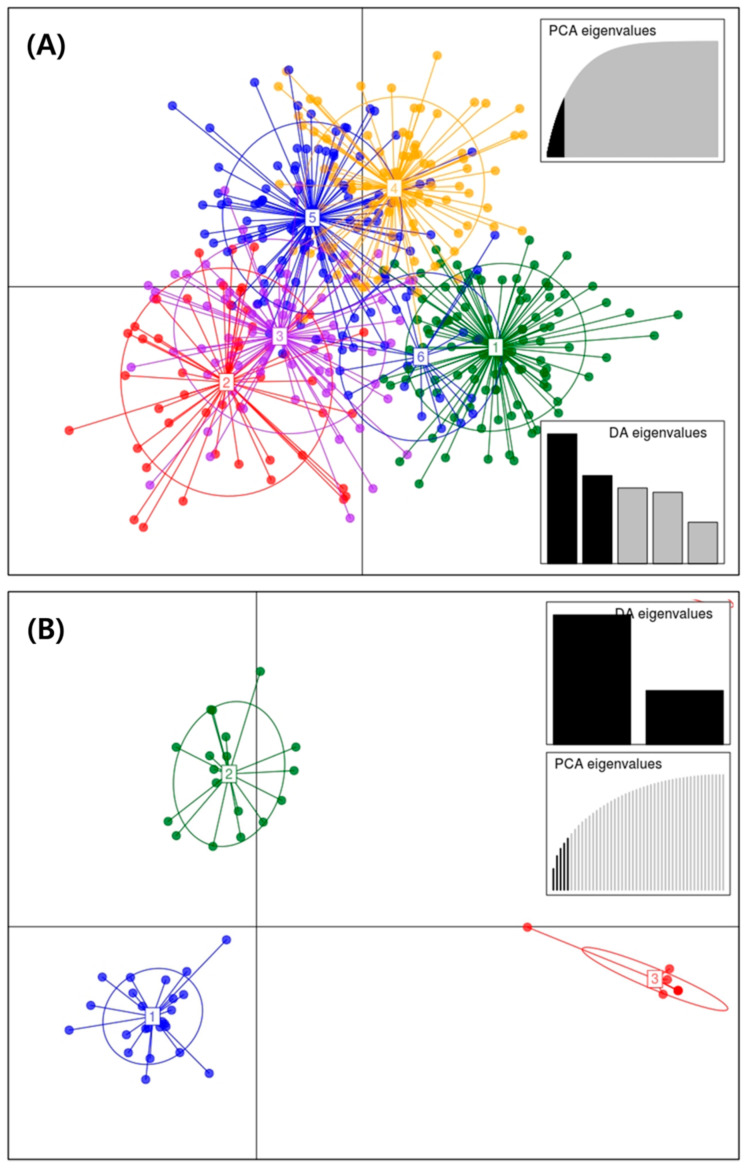
Discriminant analysis of principal components (DAPC) for 482 potato accessions. (**A**) Left six groups (C1, C2, C3, C6, C7, and C8) in Figure 1B, (**B**) Right groups (C4 and C5) in Figure 1B. Axes represent the first tow linear discriminants (LD). Circles represent groups. Dots represent individuals. Numbers represent different subpopulations identified by DAPC analysis.

To understand the pattern of the genetic structure, a Bayesian clustering analysis in STRUCTURE and a complementary ordination analysis by Discriminant Analysis of Principal Components (DAPC) was performed. STRUCTURE results suggested that the best grouping number was K = 2 based on delta K (Figure S1). Population 1 and 2 consisted of 428 and 54 accessions, respectively. Twenty-seven of 428 accessions in population 1 and 6 of 54 accessions in population 2 were genetically admixed accessions (Figure 1A). DAPC analysis was carried out using the detected number of clusters (Figure 1B). The number of detected clusters was eight, in concordance with the lowest BIC value obtained using *find.clusters* function. Twenty first PCs (50.53% of variance conserved) of PCA and seven discriminant eigenvalues were retained. These values were confirmed by cross-validation analysis. Numbers of accessions in clusters 1 to 8 were 41, 55, 85, 22, 30, 83, 80, and 86, respectively (Table 5). Diversity indices among eight clusters from results of DAPC were calculated. Results are shown in Table 5. Na and Ng ranged from 5.17 (C1) to 7.38 (C4) and from 7.63 (C5) to 13.25 (C3), respectively. Values of 1-D and H were calculated to be from 0.59 (C5) to 0.71 (C4) and from 1.18 (C1 and C5) to 1.57 (C4), respectively. Values of GD and Evenness ranged from 0.72 (C4 and C5) to 0.80 (C1 and C6) and from 0.690 (C6) to 0.802 (C8), respectively. In Fig. 1B, Linear Discriminant 1 (LD1) separated among two subpopulations (Subpopulation 1 (430 accessions) = C1, 2, 3, 6, 7, and 8; Subpopulation 2 (52 accessions) = C4 and C5), while LD2 did not separate. Based on the results of STRUCTURE, subpopulations 1 and 2 were identified to be present in populations 1 and 2, respectively. In DAPC analysis of subpopulations 1 and 2, subpopulation 2 separated three groups (26 accessions with cultivars or breeding lines, 18 accessions with wild relatives, and eight accessions with breeding lines from KOR), while subpopulation 1 did not clearly show separate groups (Figure 2A,B).

Sources of genetic differentiation were revealed among different inferred clusters by AMOVA (Table 6). Results indicated that 3% of variations could be attributed to differentiation among clusters, while 97% of variations could be attributed to differentiation within inferred clusters. PhiPT and gene flow (Nm) for 482 potato accessions were 0.026 (*p* < 0.0001) and 18.880, respectively. In subpopulation 1, 6% of variations could be attributed to differentiation among clusters, while 94% of variations could be attributed to differentiation within inferred clusters. PhiPT and Nm for subpopulation 1 were 0.056 (*p* < 0.0001) and 8.505, respectively. In subpopulation 2, 29% of variations could be attributed to differentiation among clusters while 71% of variations could be attributed to differentiation within inferred clusters. PhiPT and Nm for subpopulation 2 were 0.290 (*p* < 0.0001) and 1.224, respectively.

**Figure 3:**
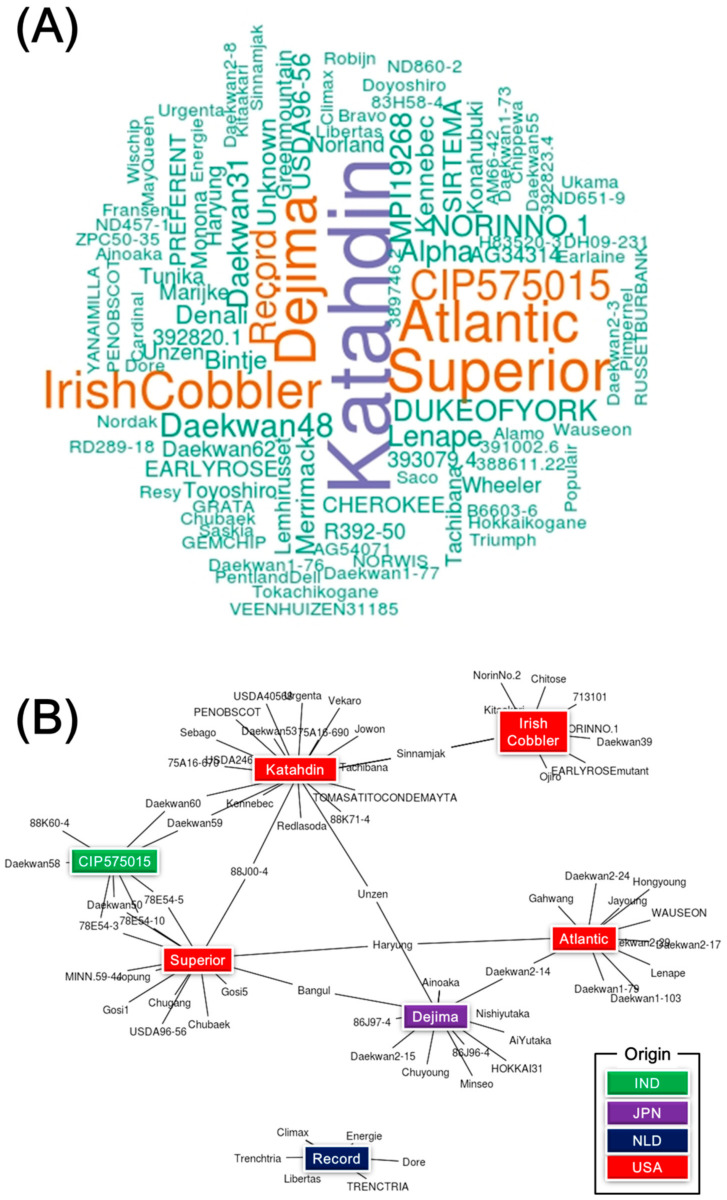
(**A**) Word cloud of 248 potato cultivars or breeding lines with their pedigree information. (**B**) Network of high frequency seven potato cultivars from results of the word cloud. IND, India; JPN, Japan; NLD, Netherlands; USA, the United States of America.

Among 440 potato cultivars (251 accessions) or breeding lines (189 accessions), only pedigree information of 248 accessions was available in the GMS database (Table S1). Using the analysis of word cloud, seven potato cultivars were the most frequently used (Figure 3A). Among them, cv. Katahdin was used the most frequently (n = 20 times), followed by cvs. Superior (n = 13), Atlantic (n = 12), Dejima (n = 12), Irish Cobbler (n = 11), CIP575015 (n = 8), and Record (n = 7) (Figure 3B). Four (cvs. Katahdin, Superior, Atlantic, and Irish Cobbler) of seven potato cultivars were from the United States of America, while the other three were from Japan, Netherlands, and India, respectively.

## 3. Discussion

Previous studies in many countries have investigated the genetic diversity of potato germplasm using SSR markers for breeding purposes and future germplasm management programs [29,36,39,40]. However, there have been few studies on the genetic diversity of potato germplasm in South Korea. In the Web of Science database, 288 of 11,488 papers of potato research between 1989 and 2019 have been published from South Korea. Of them, only one article is related to the genetic diversity of potato germplasm [27]. Our present study is the first paper to investigate genetic diversity using 24 SSR markers for 482 potato accessions conserved in NAC of South Korea. Understanding the diversity of plant germplasm is important because it provides opportunity for plant breeders to develop new and improved cultivars with desirable characteristics, including both farmer-preferred traits (yield potential and large seed, etc.) and breeder-preferred traits (pest and disease resistance and photosensitivity, etc.) [41].

In our study, a total of 257 alleles with 4 to 20 alleles (average of 10.71) per loci were detected (Table 1). Previous studies have reported 174 alleles (average of 5.8 alleles per loci) for 292 diverse genotypes of potato using 30 SSR markers [38], 249 alleles (average of 12.45 alleles per loci) of 217 Chinese potato cultivars using 20 SSR markers [28], and 190 alleles (average of 9.5 alleles per loci) of 288 potato germplasm using 20 SSR markers [26]. Reasons for such different results obtained in these studies were mainly due to different sources of potato collections [42] as well as differences in the application of marker type and the platform used for resolution of amplified products [43]. Although the number of SSR markers used in the present study was similar than other studies, it seemed that higher allele-richness than other studies was due to the composition of potato germplasm collected from various countries.

Wang et al. reported that the Shannon–Wiener index (H) and Nei’s gene diversity (GD) are reliable measures among the parameters for assessing genetic diversity [44]. The Shannon index, sometimes referred to as the Shannon–Wiener Index or the Shannon–Weaver Index, is one of several indices used to measure diversity in categorical data. It is simply the information entropy of the distribution, treating species as symbols and their relative population sizes as probabilities [44]. In this study, the I for USA germplasm (1.51) was higher than other germplasms (1.28 (CHN) to 1.38 (PER). GD for the USA germplasm (0.70) was also higher than other germplasms. This may be because the USA conserves many potato accessions, including wild species, among the other countries in this study. According to the FAO (2010) report, although the USA has fewer total potato accessions than Peru, they have more wild species (65) than Peru (only 2) [45].

Among the 482 potato germplasm accessions used in this study and conserved at NAC, 96.1% represented breeding lines (189 accessions, 39.2%), cultivars (251 accessions, 52.1%), and landraces (23 accessions, 4.8%), while only 19 accessions (3,9%) were crop wild relatives (Table S1). The last global strategy for ex situ conservation of potato analyzed 23 global potato collections, which collectively maintained 58,964 potato accessions, of which 41.2% were cultivars, breeding lines, or hybrids [46]. This summary states that gene banks in Latin America contain principally native cultivars while those in Europe, North America, and Asia contain modern cultivars, breeding materials, and wild relatives. In Asia, China (cultivars or breeding lines, 82.4%) and Japan (cultivars or breeding lines, 91.8%) showed similar compositions of potato accessions as South Korea. Some global potato gene banks in Europe and North America also showed high rates of cultivars or breeding lines among their potato accessions [46]. 

In this study, STRUCTURE and DAPC were used to analyze the population structure of 482 potato accessions to provide complementary information. Results of STRUCTURE and DAPC divided them into two subpopulations, although 482 potato accessions were separated into eight clusters in more detail by DAPC (Figure 1). In addition, subpopulation 2 from DAPC was again divided into three clusters according to their genotype or species (Figure 2B). The DAPC method provides an interesting alternative to STRUCTURE software as it does not require populations to be in HW equilibrium. In addition, it can handle large sets of data without using parallel processing software [47]. A previous study [48] has mentioned that DAPC analysis can divide the population into well-defined clusters associated with provenance, ploidy, taxonomy, and breeding program of genotypes related to their genetic structure. One study [49] has reported that DAPC is much better than STRUCTURE as it can lead to a better separation among potato germplasms. One study [43] has also reported that DAPC analysis provides a more detailed clustering for cherry populations compared to STRUCTURE analysis.

PhiPT is a measure of population differentiation due to genetic structure [50]. A PhiPT value greater than 0.15 can be considered as significant in differentiating populations [51]. In this study, a low PhiPT value in STRUCTURE (0.031) and DAPC (0.026) was found (Table 3 and Table 6), indicating a low genetic differentiation among these subpopulations in each analysis. [52] reported that an Nm value less than one indicates limited gene exchange among populations, while in our study, the Nm values of 15.828 (STRUCTURE) and 18.880 (DAPC) were high, suggesting that a high genetic exchange or high gene flow may occur and lead to a low genetic differentiation among populations [53].

Subpopulation I consisted of 430 potato accessions, of which 94.4% were breeding lines or cultivars (Table S1). Although there were not enough pedigree information or breeding program of them, a few potato cultivars might have been used to develop new potato varieties among conserved potato accessions of NAC. Results of the word cloud and network analysis showed that the number of high-frequency potato accessions was seven (Figure 3). Among them, four potato varieties (cvs. Atlantic, Katahdin, Irish cobbler, and Superior) were from the USA, and one variety (cv. Dejima) was from JPN. They came to Korea through Japan [6]. In the case of Korea, six potato accessions (except cv. Record) have been used highly frequently to develop new varieties. For example, cvs. Haryung (developed in 2006), Bangul (2011), and Suji (2019) were derived from Superior x Atlanntic, Superior x Dejima, and Banggul x Saebong, respectively (Table S1). Similar result has been reported previously, showing that cv. Katahdin, the most frequent parental in their potato population, contributes to the genetic background of all groups in a previous study [48]. One study [54] has also reported that some old parents such as Katahdin are often used extensively. Previous studies have reported that the genetic base of modern cultivated potato is very narrow [1,48,55,56,57,58]. One study [40] has reported that the most important reasons for such narrow gene base of potato varieties in USDA are the selection practiced for those characters that are desired and needed in modern varieties and the partial or complete pollen sterility present in many *Tuberosum* parents, which apparently is the result of their cytoplasm. Landraces and crop wild relatives harbor ample genetic diversity. Hence, these are valuable sources of variation for genetic enhancement and crop improvement. Their effective collection, characterization, conservation, and use will be an important asset for future sustainable crop production and adaptation under climate change scenarios [57].

Subpopulations II contained 52 potato accessions clearly separated into three groups, of which 18 accessions were wild relatives (Table S1 and Figure 2B). Among them, 26 accessions were cultivars or breeding lines from JPN, KOR, NLD, PER, and USA. However, information such as pedigree and breeding program was unavailable for them except for IT231789. ‘Inca-NO-Mezame’ (IT231789) from JPN was derived from W 822229-5 (a cross between haploid *Solanum tuberosum* spp. *tuberosum* cv. Katahdin and *S. phureja*) x P 10173-5 (a cross between 2 haploids of *S. tuberosum* ssp. *andigena*) [59]. The other eight accessions (BW15-01. BW15-05, BW15-06, BW15-07, BW15-08, BW15-09, BW15-09, and LB15-04) were from KOR. They were developed using somatic hybrids between *S. commersonii* ssp. *commersonii* Dunal PI320266 clone LZ3.2 and PT56, a dihaploid from *S. tuberosum* cv Superior (US-13122) to select four lines, HA06-1, HA06-2, HA06-4, and HA06-9, as potential parents for bacterial wilt resistance. According to GMS database, eight accessions were derived from HA06-9 x *S. tuberosum* cv. Dejima, of which seven accessions showed highly bacterial wilt resistance and one accession (IT301464) showed high potato blight resistance [60]. Crop wild relatives conserved in gene banks offer ‘an enormous and unimaginable potential’ for the discovery of valuable and desired traits [56]. Previous studies have reported that wild relatives of potato show adaptation to a wide range of habitats from sea level to 4500 m. These adaptations to a wide range of habitats have made crop wild relatives tolerant to different environmental stresses and resistant to a broad range of pests and diseases and other agricultural traits of interest [11,56,61,62,63,64]. The incorporation of novel traits from wild relatives of potatoes constitutes a relatively minor but important effort of breeding programs worldwide because germplasm enhancement is needed whenever desirable traits or alleles are not present in accessible, cultivated materials [55]. 

In this study, a global collection of 482 potato accessions was genotyped with 24 SSR markers to evaluate their genetic diversity and population structure. These germplasms accessions showed a high level of genetic diversity among all molecular marker loci. They were assigned into two major subpopulations based upon population structure and DAPC analysis. Accessions of subpopulation 1 mainly consisted of cultivars and breeding lines. Accessions of subpopulations 2 basically corresponded to wild relatives of potatoes. In addition, subpopulation 1 showed a narrow genetic base. By selecting parents that are genetically similar, breeders often restrict the amount of genetic variation that can be evaluated in the offspring, meaning opportunities lost in terms of utilization of available genetic resources. In its worst manifestations, it can lead to a progressively narrower genetic base, slower progress (genetic gain), and increased risk of crop vulnerability because there is a tendency to give more attention to adaptation through selection than to the generation of new variability or the maintenance of adaptability [65]. Therefore, the collection and preservation of plant genetic resources are of immense importance for crop breeding to support the demands of a growing human population. Effective management and utilization of plant genetic resources require information about the origin of genotypes, phenotypic traits, and genetic diversity (identified by molecular techniques) [66]. Results of this study provide useful information for potato improvement and conservation programs, although further studies are needed to make an accurate evaluation of genetic diversity and phenotypic traits.

## 4. Materials and Methods

### 4.1. Plant Materials

A total of 482 potato accessions were obtained from the National Agrobiodiversity Center (NAC) at the Rural Development Administration in South Korea (Table S1).

### 4.2. DNA Extraction 

Genomic DNA was extracted from potato leaves using a Qiagen DNA extraction kit (Qiagen, Hilden, Germany). DNA quality and quantity were measured using 1% (*w*/*v*) agarose gel and spectrophotometry (Epoch, BioTek, Winooski, VT, USA). Extracted DNA was diluted to 30 ng/uL and stored at −20 °C until further PCR amplification.

### 4.3. SSR Genotyping

For SSR analysis, a total of 24 SSRs designed by Ghistain et al. [36] were fluorescently labeled (6-FAM, HEX, and NED) and used for the detection of amplification products (Table S2). PCR reactions were carried out using 25 ul reaction mixture containing 30 ng template DNA, 1.5 mM MgCl2, 0.2 mM of each dNTPs, 0.5 um of each primer, and 1 U *Taq* polymerase (Inclone, Korea). The amplification was performed with the following cycling conditions: Initial denaturation at 94 °C for 5 min, followed by 35 cycles of denaturation at 95 °C for 30 s, annealing at 55 to 61 °C for 30 s, extension at 72 °C for 1 min, and a final extension step at 72 °C for 10 min. Each amplicon was resolved on ABI prism 3500 DNA sequence (ABI3500, Thermo Fisher Scientific Inc., Wilmington, DE, USA) and scored using a Gene Mapper Software (Version 4.0, Thermo Fisher Scientific Inc.).

### 4.4. Population Structure and Genetic Diversity

Null allele frequencies of 24 SSR markers were estimated using the poppr package for R software (Table S3) [63]. The number of alleles (Na), number of genotypes (Ng), Shannon–Wiener index (H) equation:

$$H=-\overset{S}{\underset{i=1}{\sum }}p_{i}\log_{2}p_{i}$$

when *p_i_* is the proportion of genotype *I* and *S* is the number of genotypes. Nei’s gene diversity (GD) equation:

$$h=\left(\frac{n}{n-1}\right)1-\overset{k}{\underset{i=1}{\sum }}p_{i}^{2}$$

$$GD=\overset{m}{\underset{l=1}{\sum }}\frac{h_{l}}{m}\;$$

when *p_i_* is the population frequency of the *i*th allele in a locus, *k* is the number of alleles in a particular locus, *n* is the number of observed alleles in the population and *h_l_* is the value of *h* at the *l*th locus, and Evenness (E5) equation:

$$Evenness\left(E5\right)=\frac{\left(\frac{1}{\lambda }\right)-1}{e^{H}-1}$$

when 1/*λ* is Stoddart and taylor’s index and *H* is Shannon diversity, were calculated using poppr package for R software [67]. Analysis of molecular variance (AMOVA) and calculation of the coefficient of genetic differentiation among populations (PhiPT) and gene flow (Nm) was done using GenAlEx software (6.5 version) with 999 permutations [68]. The population structure was evaluated with a DAPC using the adegenet package for R software [69,70] according to Lee et al. [33]. Bayesian-based clustering was performed using STRUCTURE v.2.3.4 [71], testing 3 independent runs with K from 1 to 11. Each run had a burn-in period of 50,000 iterations and 500,000 Monte Carlo Markov iterations, assuming an admixture model. The output was subsequently visualized with STRUCTURE HARVESTER v.0.9.94 [72]. The most likely number of clusters was inferred according to Evanno [73]. A membership coefficient q > 0.8 was used to assign samples to clusters. Samples within a cluster with a membership coefficient ≤0.8 were considered ‘genetically admixed’.

### 4.5. Word Cloud and Network Analysis

Word cloud analysis was performed using *wordcloud* package for R software to determine the frequency of potato use. Using results of word cloud analysis, network analysis was performed using *tidygraph* package for software to identify the relationship among potato accessions.

## Figures and Tables

**Table 1:** Genetic diversity parameters of 24 simple sequence repeat (SSR) markers in 482 potato accessions.

Total	Na	Ng	H	GD	Evenness
STG0001	13	42	1.84	0.80	0.77
STM0019a,b	20	60	2.00	0.83	0.78
STG0010	8	17	1.24	0.66	0.80
STM0031	18	51	1.85	0.79	0.70
STG0016	17	53	1.78	0.80	0.80
STM0037	14	51	1.81	0.79	0.74
STG0025	6	9	0.82	0.52	0.86
STM1052	13	48	1.66	0.76	0.76
STI0001	9	29	1.61	0.77	0.85
STM1053	5	6	0.39	0.18	0.46
STI0003	16	43	1.78	0.80	0.83
STM1064	6	11	1.07	0.59	0.75
STI0004	9	34	1.51	0.73	0.77
STM1104	6	15	1.18	0.63	0.74
8STI0012	13	36	1.63	0.76	0.76
STM1106	15	26	1.30	0.61	0.57
9STI0014	7	15	1.18	0.65	0.81
STM5114	11	22	1.62	0.76	0.78
10STI0030	10	31	1.41	0.70	0.75
STM5121	4	8	0.85	0.50	0.73
11STI0032	9	27	1.75	0.81	0.91
STM5127	9	41	1.73	0.78	0.77
12STI0033	10	33	1.62	0.77	0.81
STPoAc58	9	19	0.80	0.38	0.49
mean	10.71	30.29	1.44	0.68	0.75

Na: Number of observed alleles; Ng: Number of genotypes; H: Shannon–Wiener index; GD: Nei’s gene diversity.

**Table 2:** Genetic diversity parameters of seven origins in 482 potato accessions using 24 SSRs.

Origin	N	Na	Ng	H	GD	Evenness
BGR	29	5.46 ± 1.86	9.00 ± 4.33	1.30	0.67	0.79
CHN	42	5.50 ± 2.21	9.79 ± 5.13	1.28	0.66	0.82
JPN	48	5.83 ± 2.16	11.00 ± 5.63	1.35	0.68	0.80
KOR	122	6.29 ± 2.31	14.50 ± 7.96	1.33	0.66	0.78
NLD	60	5.88 ± 2.19	11.29 ± 5.80	1.29	0.66	0.79
PER	41	6.08 ± 2.15	11.46 ± 5.84	1.38	0.68	0.77
USA	140	9.88 ± 3.57	21.88 ± 10.3	1.51	0.70	0.74

Na: Number of observed alleles; Ng: Number of genotypes; H: Shannon-Wiener index; GD: Nei’s gene diversity; BGR: Bulgaria; CHN: China; JPN: Japan; KOR: South Korea; NLD: Netherlands; PER: Peru; USA: United States of America.

**Table 3:** Analysis of molecular variance (AMOVA) within and among potato populations based on 24 SSR data according to their seven origins.

SV	Df	SS	MS	Est. Var.	%	PhiPT	Nm
Among origin	6	481.774	80.296	0.832	3%	0.031 ***	15.828
Within origin	475	12,506.242	26.329	26.329	97%		
Total	481	12,988.017		27.161	100%		

SV: Source of variation; Df: Degrees of freedom; SS: Sum of squares; MS: Mean square; Est. Var.: Estimated variance; %: Percentage of variation; Nm: Gene flow; *** *p* < 0.001.

**Table 4:** Pairwise population PhiPT values (above diagonal) and Nm values (below diagonal) based on 999 permutations from AMOVA according to seven origins (all PhiPT values were significantly greater than 0, *p* < 0.05).

	BGR	CHN	JPN	KOR	NLD	PER	USA
BGR	-	0.018	0.028	0.040	0.023	0.062	0.024
CHN	27.334	-	0.008	0.015	0.013	0.083	0.017
JPN	17.609	63.589	-	0.009	0.019	0.080	0.014
KOR	12.143	31.996	52.507	-	0.032	0.095	0.015
NLD	21.566	36.647	25.911	15.165	-	0.076	0.020
PER	7.628	5.494	5.768	4.775	6.041	-	0.060
USA	20.009	29.776	34.302	31.760	24.408	7.897	-

BGR: Bulgaria; CHN: China; JPN: Japan; KOR: South Korea; NLD: Netherlands; PER: Peru; USA: United States of America.

**Table 5:** Genetic diversity parameters of eight populations from DAPC.

Cluster	N	Na	Ng	H	GD	Evenness
C1	41	5.17 ± 2.28	8.00 ± 4.68	1.18	0.62	0.80
C2	55	5.88 ± 2.03	10.08 ± 4.52	1.27	0.65	0.79
C3	85	6.33 ± 2.41	13.25 ± 7.10	1.31	0.66	0.77
C4	22	7.38 ± 2.76	8.71 ± 3.48	1.57	0.74	0.72
C5	30	5.33 ± 1.99	7.63 ± 3.76	1.18	0.61	0.72
C6	83	5.75 ± 2.36	11.71 ± 6.93	1.28	0.65	0.80
C7	80	6.33 ± 2.48	12.50 ± 6.55	1.28	0.65	0.78
C8	86	6.00 ± 2.36	12.46 ± 6.76	1.29	0.65	0.78

Na: Number of observed alleles; Ng: Number of genotypes; H: Shannon–Wiener index; GD: Nei’s gene diversity.

**Table 6:** Analysis of molecular variance (AMOVA) within and among potato populations based on 24 SSR data according to results of DAPC analysis

Population	SV	DF	SS	MS	Est. Var.	%	PhiPT	Nm
Total	Among Pops	7	472.542	67.506	0.699	3%	0.026 ***	18.880
	Within Pops	474	12,515.475	26.404	26.404	97%		
	Total	481	12,988.017		27.103	100%		
Sub-population 1	Among Pops	5	626.882	125.376	1.454	6%	0.056 ***	8.505
	Within Pops	424	10,489.416	24.739	24.739	94%		
	Total	429	11,116.298		26.194	100%		
Sub-population 2	Among Pops	2	318.496	159.248	8.742	29%	0.290 ***	1.224
	Within Pops	49	1048.524	21.398	21.398	71%		
	Total	51	1367.019		30.140	100%		

SV: Source of variation; DF: Degrees of freedom, SS: Sum of squares; MS: Mean square; Est. Var.: Estimated variance; %: Percentage of variation; Nm: Gene flow; *** *p* < 0.001.

## Supplementary Materials

The following are available online at https://www.mdpi.com/article/10.3390/plants10040752/s1, Figure S1: Estimation of population using LnP(D) derived ΔK. Delta K values for different numbers of populations (K) assumed in analysis completed with the STRUCTURE software, Table S1: List of 482 potato accessions used in this study, Table S2: List of 224 SSR markers used in this study, Table S3: Missing rate (%) of 24 SSR markers in this study.
