# Supplementary material for: Genetic Diversity and Population Structure of Potato Germplasm in RDA-Genebank: Utilization for Breeding and Conservation
Source: Plants (Basel). 2021 Apr 12;10(4):752. doi: 10.3390/plants10040752 (PMC8068792; doi:10.3390/plants10040752)
Supplement: Supplementary file 1 [file plants-10-00752-s001.pdf]

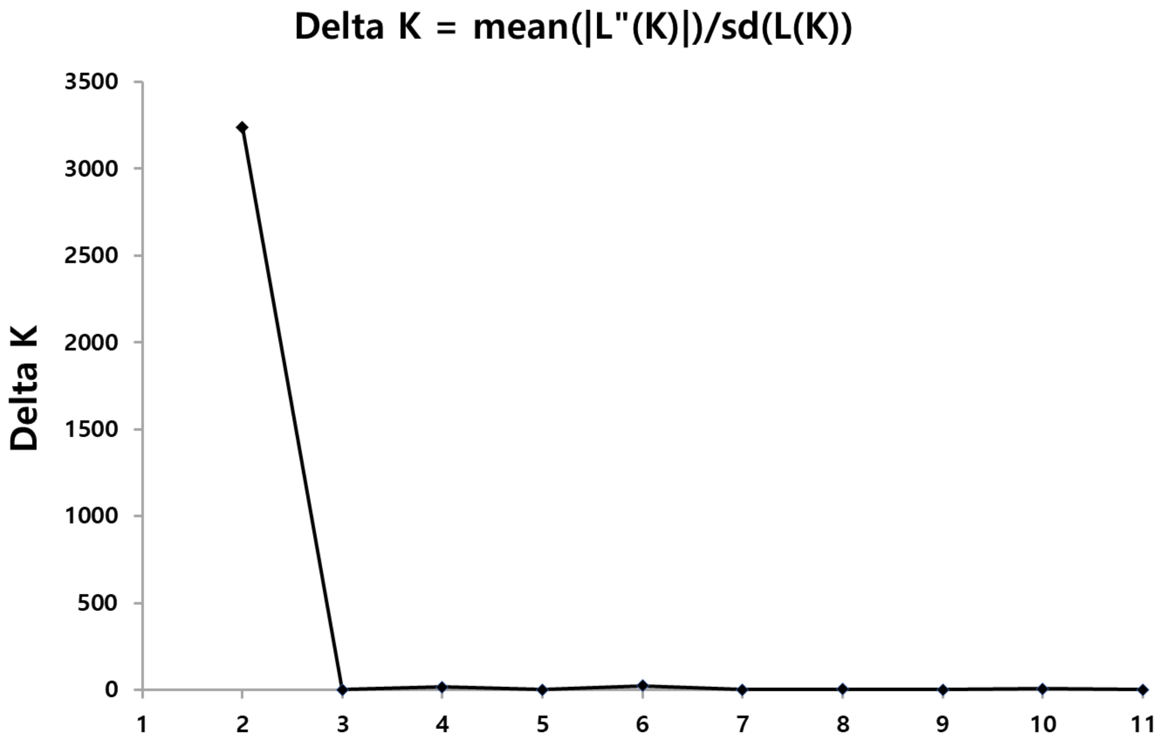

Figure S1. Estimation of population using LnP(D) derived ΔK. Delta K values for different numbers of populations (K) assumed in analysis completed with the STRUCTURE software.

Table S1. List of 482 potato accessions used in this study

IT No.	Origin	Scientic Name	Name	Type	Pedigree	ST	DAPC
IT109171	KOR	Solanum tuberosum subsp. tuberosum	CI-31	Landrace		1	7
IT231562	USA	Solanum tuberosum subsp. tuberosum	Abnaki	Cultivar	USDA 1276-185 x USDA B 4116-2	1	6
IT231563	NLD	Solanum tuberosum subsp. tuberosum	Ajax	Cultivar	Froma * MPI19268	1	7
IT231565	USA	Solanum tuberosum subsp. tuberosum	AK Red	Cultivar		1	3
IT231566	USA	Solanum tuberosum subsp. tuberosum	Aramo	Cultivar	Merimack*Kennebec	1	6
IT231567	USA	Solanum tuberosum subsp. tuberosum	Allagash Russet	Cultivar	BR 7093-56 x USDA B 6042-3	2	8
IT231568	USA	Solanum tuberosum subsp. tuberosum	ALLEGANY	Cultivar	M 297-17 x pollenmixture	1	2
IT231569	NLD	Solanum tuberosum subsp. tuberosum	Alpha	Cultivar	Paul kruger * Preferent	1	3
IT231570	NLD	Solanum tuberosum subsp. tuberosum	Alpha	Cultivar		1	7
IT231574	NLD	Solanum tuberosum subsp. tuberosum	Akula	Cultivar	Axila*(Saskia*Schwalbe)	1	6
IT231577	NLD	Solanum tuberosum subsp. tuberosum	Avon	Cultivar	(USDA X96-56)*1464-26	1	2
IT231582	NLD	Solanum tuberosum subsp. tuberosum	Bintje	Cultivar	Mustersen * Fransen	2	8
IT231584	NLD	Solanum tuberosum subsp. tuberosum	Cardinal	Cultivar	TULNER/DE VRIES 54-30-8 x SVP 55-89	1	8
IT231586	USA	Solanum tuberosum subsp. tuberosum	CHEROKEE	Cultivar	USDA 96-56 x USDA 528-170	1	6
IT231587	JPN	Solanum tuberosum subsp. tuberosum	Chitose	Cultivar	IRISH COBBLER x HONIKU 393	1	2
IT231588	NLD	Solanum tuberosum subsp. tuberosum	CLEOPATRA	Cultivar	ZPC50-35 * Desiree	1	2
IT231593	NLD	Solanum tuberosum subsp. tuberosum	Corine	Cultivar	SIRTEMA x Y 193(Katahdin)	1	1
IT231596	USA	Solanum tuberosum subsp. tuberosum	Denali	Cultivar	LENAPE x ALASKA 1-62-90-64	1	6
IT231597	NLD	Solanum tuberosum subsp. tuberosum	Desiree	Cultivar	Urgenta * Depesche	1	7
IT231598	NLD	Solanum tuberosum subsp. tuberosum	Diamant	Cultivar	Cardinal mutant	1	7
IT231599	JPN	Solanum tuberosum subsp. tuberosum	Touya	Cultivar	R392-50 x WB 77025-2	1	3
IT231600	JPN	Solanum tuberosum subsp. tuberosum	Toyoshiro	Cultivar	HOKKAI 19 x ENIWA	1	6
IT231601	NLD	Solanum tuberosum subsp. tuberosum	Eigenheimer	Cultivar	Blauwe Reuzen * Fransen	1	6
IT231608	USA	Solanum tuberosum subsp. tuberosum	GEMCHIP	Cultivar	BR 5960-9 x ND 5737-3	1	6
IT231609	USA	Solanum tuberosum subsp. tuberosum	Gigant	Cultivar	Elvira * AM66-42	1	6
IT231610	NLD	Solanum tuberosum subsp. tuberosum	GRANOLA	Cultivar	3333-60 * 269-04	1	6
IT231612	NLD	Solanum tuberosum subsp. tuberosum	Helena	Cultivar	H1152/76 * Agria	1	7
IT231613	NLD	Solanum tuberosum subsp. tuberosum	Hertha	Cultivar	Dijkhuis 61-133-3 * Konst 62-374	1	3
IT231615	JPN	Solanum tuberosum subsp. tuberosum	Hokkaikogane	Cultivar	TOYOSHIRO x HOKKAI 51	1	8
IT231622	USA	Solanum tuberosum subsp. tuberosum	Katahdin	Cultivar	USDA 40568 x USDA 24642	1	7
IT231623	USA	Solanum tuberosum subsp. tuberosum	Kennebec	Cultivar	(Chippewa*Katahdin)*(USDA 3895-13*Earlaine)	1	6
IT231625	JPN	Solanum tuberosum subsp. tuberosum	Konafubuki	Cultivar	TOYOSHIRO x WB 66201-10	1	7
IT231626	NLD	Solanum tuberosum subsp. tuberosum	Kondor	Cultivar	Konst 61333 * Wilja	1	2

IT231628	USA	Solanum tuberosum subsp. tuberosum	Lachipper	Cultivar	GREEN MOUNTAIN x CAYUGA	1	6
IT231630	NLD	Solanum tuberosum subsp. tuberosum	Record	Cultivar	Trenchtria * Energie	1	7
IT231631	USA	Solanum tuberosum subsp. tuberosum	Lemihi Russet	Cultivar	PIONEER x A 63126-8	1	7
IT231632	NLD	Solanum tuberosum subsp. tuberosum	Libertas	Cultivar	RECORD x VEENHUIZEN 31185	1	7
IT231638	JPN	Solanum tuberosum subsp. tuberosum	May Queen	Cultivar		1	2
IT231639	NLD	Solanum tuberosum subsp. tuberosum	Mirca	Cultivar	Triumph * Kerkov B/53	1	7
IT231641	USA	Solanum tuberosum subsp. tuberosum	Monona	Cultivar	Katahdin selfed * Chippewa selfed	1	6
IT231645	JPN	Solanum tuberosum subsp. tuberosum	Nishiyutaka	Cultivar	DEJIMA x CHOUKEI 65	1	6
IT231648	USA	Solanum tuberosum subsp. tuberosum	Nordak	Cultivar	ND 457-1 x ND 457-1	1	6
IT231649	USA	Solanum tuberosum subsp. tuberosum	Norgold Russet	Cultivar	A 119-1 x ND 2475-8	1	7
IT231650	USA	Solanum tuberosum subsp. tuberosum	Norking Russet	Cultivar	OOKSACK x ND 9567-2R	1	8
IT231651	USA	Solanum tuberosum subsp. tuberosum	Norland	Cultivar	ND 626 x REDKOTE	1	6
IT231652	JPN	Solanum tuberosum subsp. tuberosum	Ojiro	Cultivar	IRISH COBBLER x NORIN NO.1	1	7
IT231654	USA	Solanum tuberosum subsp. tuberosum	PENOBSCOT	Cultivar	USDA 927-3 x KATAHDIN	1	6
IT231658	USA	Solanum tuberosum subsp. tuberosum	Pentland Dell	Cultivar	Roslin Chania * Roslin Sasamua	1	7
IT231661	USA	Solanum tuberosum subsp. tuberosum	Pimpernel	Cultivar	POPULAIR x MULDER K 101	1	7
IT231665	NLD	Solanum tuberosum subsp. tuberosum	Radosa	Cultivar	BINTJE x MPI 19268	1	7
IT231666	NLD	Solanum tuberosum subsp. tuberosum	Present	Cultivar	LANDSKROON x JAUNE D'OR	1	1
IT231667	USA	Solanum tuberosum subsp. tuberosum	Red lasoda	Cultivar	Mutant of Lasoda (Triumph * Katahdin)	1	6
IT231669	USA	Solanum tuberosum subsp. tuberosum	RED PONTIAC	Cultivar	PONTIAC mutant	1	3
IT231671	USA	Solanum tuberosum subsp. tuberosum	Red warba	Cultivar	Warbar muatnt	1	6
IT231673	NLD	Solanum tuberosum subsp. tuberosum	Resy	Cultivar	SVP 50-2017 x MPI 19268	1	1
IT231674	USA	Solanum tuberosum subsp. tuberosum	Rosa	Cultivar	Wauseon * J171-8	1	3
IT231676	USA	Solanum tuberosum subsp. tuberosum	RUSSET BURBANK	Cultivar	BURBANK mutant ?	1	2
IT231677	USA	Solanum tuberosum subsp. tuberosum	RUSSET NORKOTAH	Cultivar	ND 9526-4R x ND 9687-5R	1	2
IT231680	JPN	Solanum tuberosum subsp. tuberosum	Samwonseo	Cultivar	TPS of Old jersey peachblow	1	6
IT231681	NLD	Solanum tuberosum subsp. tuberosum	Sandra	Cultivar	MULTA x VTN 62-33-3	1	6
IT231684	JPN	Solanum tuberosum subsp. tuberosum	Sayaka	Cultivar	Pentland dell * R392-50	1	1
IT231685	USA	Solanum tuberosum subsp. tuberosum	Sebago	Cultivar	CHIPPEWA x KATAHDIN	1	7
IT231686	JPN	Solanum tuberosum subsp. tuberosum	Setoyutaka	Cultivar	SAIKAI 10 x UNZEN	1	2
IT231688	JPN	Solanum tuberosum subsp. tuberosum	Shimabara	Cultivar	NORIN NO.1 x GINEKE	1	2
IT231694	NLD	Solanum tuberosum subsp. tuberosum	Spunta	Cultivar	Bea * USDA96-56	1	6
IT231695	JPN	Solanum tuberosum subsp. tuberosum	Tachibana	Cultivar	NORIN NO.1 x KATAHDIN	1	6
IT231696	JPN	Solanum tuberosum subsp. tuberosum	Tahume	Cultivar		1	3
IT231698	USA	Solanum tuberosum subsp. tuberosum	Teton	Cultivar	USDA 45146 x EARLAINE	1	6

IT231702	JPN	Solanum tuberosum subsp. tuberosum	Tunika	Cultivar	Lu56.186/21N * Lu56.183/2	1	7
IT231703	JPN	Solanum tuberosum subsp. tuberosum	Unzen	Cultivar	NORIN NO.1 x KATAHDIN	1	1
IT231705	JPN	Solanum tuberosum subsp. tuberosum	Wadasaki	Cultivar		1	1
IT231707	JPN	Solanum tuberosum subsp. tuberosum	Waseshiro	Cultivar	KONKEI 7 x HOKKAI 39	1	2
IT231708	JPN	Solanum tuberosum subsp. tuberosum	Wheeler	Cultivar	Introduced to Japan from USA by 125-4	1	6
IT231710	USA	Solanum tuberosum subsp. tuberosum	Wischip	Cultivar	WIS 55-306.58 x W 231	1	6
IT231713	JPN	Solanum tuberosum subsp. tuberosum	Yukijiro	Cultivar	KENNEBEC x NORIN NO.2	1	2
IT231714	JPN	Solanum tuberosum subsp. tuberosum	Kitaakari	Cultivar	IRISH COBBLER x TUNIKA	1	2
IT231717	PER	Solanum tuberosum subsp. tuberosum	CANCHAN	Cultivar	BL-1.2 x MURILLO III-80	1	6
IT231741	PER	Solanum tuberosum subsp. tuberosum	TOMASA TITO CONDEMAYTA	Cultivar	(USDA B 606 x KATAHDIN) x (RENACIMIENTO (adg) x YANA IMILLA)	2	6
IT231742	USA	Solanum tuberosum subsp. tuberosum	Snowden	Cultivar	LENAPE x WISCHIP	1	3
IT231743	USA	Solanum tuberosum subsp. tuberosum	Norwis	Cultivar	RD 289-18 x MONONA	1	6
IT231746	CHN	Solanum tuberosum subsp. tuberosum	Favorita	Cultivar	ZPC 50-35 * ZPC 55-37	1	7
IT231747	CHN	Solanum tuberosum subsp. tuberosum	Jo Dae-baek	Cultivar		1	7
IT231749	CHN	Solanum tuberosum subsp. tuberosum	Rus3	Cultivar		1	3
IT231750	CHN	Solanum tuberosum subsp. tuberosum	Jimba	Cultivar		1	7
IT231751	CHN	Solanum tuberosum subsp. tuberosum	Safflower	Cultivar		1	2
IT231752	CHN	Solanum tuberosum subsp. tuberosum	Unknown	Cultivar		1	7
IT231757	USA	Solanum tuberosum subsp. tuberosum	Leice	Cultivar		1	2
IT231758	USA	Solanum tuberosum subsp. tuberosum	Unica	Cultivar	387521.3 x APHRODITE	2	8
IT231765	CHN	Solanum tuberosum subsp. tuberosum	RN-2	Cultivar		1	1
IT231766	CHN	Solanum tuberosum subsp. tuberosum	RN-3	Cultivar		1	7
IT231767	CHN	Solanum tuberosum subsp. tuberosum	RN-4	Cultivar		1	1
IT231768	CHN	Solanum tuberosum subsp. tuberosum	RN-5	Cultivar		1	7
IT231769	CHN	Solanum tuberosum subsp. tuberosum	RN-6	Cultivar		1	1
IT231770	CHN	Solanum tuberosum subsp. tuberosum	RN-8	Cultivar		1	3
IT231771	CHN	Solanum tuberosum subsp. tuberosum	RN-9	Cultivar		1	3
IT231772	CHN	Solanum tuberosum subsp. tuberosum	RN-10	Cultivar		1	1
IT231773	CHN	Solanum tuberosum subsp. tuberosum	RN-11	Cultivar		1	2
IT231774	CHN	Solanum tuberosum subsp. tuberosum	RN-12	Cultivar		1	2
IT231775	CHN	Solanum tuberosum subsp. tuberosum	Habirob-1	Cultivar		2	8
IT231776	CHN	Solanum tuberosum subsp. tuberosum	Habirob-2	Cultivar		2	8
IT231777	CHN	Solanum tuberosum subsp. tuberosum	Habirob-5	Cultivar		1	7
IT231778	CHN	Solanum tuberosum subsp. tuberosum	Habirob-6	Cultivar		1	7
IT231779	CHN	Solanum tuberosum subsp. tuberosum	Habirob-7	Cultivar		1	6

IT231780	CHN	Solanum tuberosum subsp. tuberosum	Habirob-8	Cultivar		2	8
IT231781	CHN	Solanum tuberosum subsp. tuberosum	Habirob-9	Cultivar		1	7
IT231782	CHN	Solanum tuberosum subsp. tuberosum	Habirob-10	Cultivar		1	7
IT231784	CHN	Solanum tuberosum subsp. tuberosum	Habirob-12	Cultivar		1	3
IT231785	CHN	Solanum tuberosum subsp. tuberosum	Habirob-13	Cultivar		1	2
IT231786	CHN	Solanum tuberosum subsp. tuberosum	Habirob-14	Cultivar		1	7
IT231787	CHN	Solanum tuberosum subsp. tuberosum	Habirob-15	Cultivar		1	7
IT231788	CHN	Solanum tuberosum subsp. tuberosum	Mummy	Cultivar		1	7
IT231789	JPN	Solanum tuberosum subsp. tuberosum	Inca-No-Mezame	Cultivar	W 822229-5 x P 10173-5	2	5
IT231791	NLD	Solanum tuberosum subsp. tuberosum	Ailsa	Cultivar	G.4324(545) * Maris Piper	1	7
IT231792	NLD	Solanum tuberosum subsp. tuberosum	Asterix	Cultivar	Cardinal * SVP Ve 709	1	7
IT231794	NLD	Solanum tuberosum subsp. tuberosum	Eva	Cultivar	DUKE OF YORK * Bato	1	7
IT231795	NLD	Solanum tuberosum subsp. tuberosum	Elkana	Cultivar	Mara * Prominent	1	3
IT231796	NLD	Solanum tuberosum subsp. tuberosum	Escort	Cultivar	Rental * Cebeco 64-197-16	1	7
IT231797	NLD	Solanum tuberosum subsp. tuberosum	Estima	Cultivar	Oldenburger 51-640 * G3014	1	2
IT231798	NLD	Solanum tuberosum subsp. tuberosum	Famosa	Cultivar	ESTIMA mutant	1	2
IT231800	NLD	Solanum tuberosum subsp. tuberosum	Nena	Cultivar	GRANDIFOLIA x 52/72/2206	1	7
IT231801	NLD	Solanum tuberosum subsp. tuberosum	Populair	Cultivar	ROBIJN x MONOPOOL	1	1
IT231802	NLD	Solanum tuberosum subsp. tuberosum	Robijn	Cultivar	RODE STAR x PREFERENT	1	1
IT231803	NLD	Solanum tuberosum subsp. tuberosum	Tanja	Cultivar	DUKE OF YORK x ROPTA Y 14 F	1	7
IT231804	NLD	Solanum tuberosum subsp. tuberosum	VENOUSKA	Cultivar	Blanka * VK 65-199	1	2
IT231805	USA	Solanum tuberosum subsp. tuberosum	Bake-King	Cultivar	GREEN MOUNTAIN x MERRIMACK	1	6
IT231806	USA	Solanum tuberosum subsp. tuberosum	Belrus	Cultivar	PENOBSCOT x W 39-1	1	6
IT231807	USA	Solanum tuberosum subsp. tuberosum	Bison	Cultivar	ND 5124-1R x ND 4652-4R	1	6
IT231808	USA	Solanum tuberosum subsp. tuberosum	Early Gem	Cultivar	RUSSET BURBANK x USDA 96-56	2	8
IT231809	USA	Solanum tuberosum subsp. tuberosum	Early ohio	Cultivar	EARLY ROSE x EARLY ROSE	1	3
IT231810	USA	Solanum tuberosum subsp. tuberosum	Garnet Chili	Cultivar	ROUGH PURPLE CHILI seedling	1	7
IT231811	USA	Solanum tuberosum subsp. tuberosum	Green mountain	Cultivar	DUNMORE x EXCELSIOR	2	6
IT231812	USA	Solanum tuberosum subsp. tuberosum	Lenape	Cultivar	USDA B 3672-3 x DELTA GOLD	1	6
IT231813	USA	Solanum tuberosum subsp. tuberosum	Ontario	Cultivar	JUBEL x USDA 44537	1	6
IT231814	USA	Solanum tuberosum subsp. tuberosum	Triumph	Cultivar	PEERLESS x EARLY ROSE	1	6
IT231815	USA	Solanum tuberosum subsp. tuberosum	Viking	Cultivar	Redskin * Nordak	1	6
IT231816	USA	Solanum tuberosum subsp. tuberosum	White rose	Cultivar	JACKSON seedling	1	2
IT231817	USA	Solanum tuberosum subsp. tuberosum	Early blue	Cultivar		2	8
IT231818	USA	Solanum tuberosum subsp. tuberosum	RANGER RUSSET	Cultivar	Butte * A6595-3	1	2

IT231819	USA	Solanum tuberosum subsp. tuberosum	FRONTIER RUSSET	Cultivar	A66102-16 * WN330-1	1	2
IT231820	USA	Solanum tuberosum subsp. tuberosum	Ackersegen	Cultivar	HINDENBURG x ALLERFRUHESTE GELBE	1	8
IT231822	USA	Solanum tuberosum subsp. tuberosum	Dore	Cultivar	Duke of York * (Record * (Bravo * Alpha))	1	3
IT231823	USA	Solanum tuberosum subsp. tuberosum	Ambra	Cultivar	BERBER x CAESAR	1	3
IT231824	USA	Solanum tuberosum subsp. tuberosum	Bertita	Cultivar		1	7
IT231826	USA	Solanum tuberosum subsp. tuberosum	Kojima	Cultivar	N39 * 19175	2	5
IT231827	USA	Solanum tuberosum subsp. tuberosum	Kuchia Akita	Cultivar		2	5
IT231828	USA	Solanum tuberosum subsp. tuberosum	Koyola	Cultivar		1	7
IT231829	USA	Solanum tuberosum subsp. tuberosum	Prampo	Cultivar		1	1
IT231830	USA	Solanum tuberosum subsp. tuberosum	Fina	Cultivar	2213/43 x MPI 19148	1	2
IT231831	USA	Solanum tuberosum subsp. tuberosum	Kabinika	Cultivar		2	7
IT231832	USA	Solanum tuberosum subsp. tuberosum	Climax	Cultivar	Bintje * Record	1	6
IT231833	USA	Solanum tuberosum subsp. tuberosum	Pentland Javelin	Cultivar	2693ABC(2) x 11-79	1	7
IT231835	USA	Solanum tuberosum subsp. tuberosum	Pentland Ace	Cultivar	CRAIGS DEFIANCE x 997A(44)	2	8
IT231836	USA	Solanum tuberosum subsp. tuberosum	Capiro	Cultivar		2	8
IT231837	USA	Solanum tuberosum subsp. tuberosum	Pentland Marble	Cultivar	3305(6) x 3392(1)	2	8
IT231838	USA	Solanum tuberosum subsp. tuberosum	Ukama	Cultivar	MARIJKE x SIRTEMA	1	2
IT231840	USA	Solanum tuberosum subsp. tuberosum	Anett	Cultivar	48060 x MPI 44.1016/10	1	7
IT231841	USA	Solanum tuberosum subsp. tuberosum	Anthila	Cultivar		1	7
IT231842	USA	Solanum tuberosum subsp. tuberosum	Amarillo	Cultivar		1	7
IT231843	USA	Solanum tuberosum subsp. tuberosum	Runto Huairo	Cultivar		1	7
IT231844	USA	Solanum tuberosum subsp. tuberosum	A9520-45	Cultivar		1	3
IT231845	USA	Solanum tuberosum subsp. tuberosum	A96517-2	Cultivar		1	3
IT231846	USA	Solanum tuberosum subsp. tuberosum	A97084-44	Cultivar		2	8
IT231847	USA	Solanum tuberosum subsp. tuberosum	Tamelina	Cultivar		2	5
IT231848	USA	Solanum tuberosum subsp. tuberosum	Murufurena	Cultivar		2	5
IT231851	JPN	Solanum tuberosum subsp. tuberosum	Red moon	Cultivar		1	3
IT231852	JPN	Solanum tuberosum subsp. tuberosum	ainoaka	Cultivar	DEJIMA x NORLAND	2	2
IT231853	USA	Solanum tuberosum subsp. tuberosum	Dakota Peal	Cultivar	ND 1118-1 x ND 944-6	1	7
IT231862	USA	Solanum tuberosum subsp. tuberosum	Yukon Gold	Cultivar	NORGLEAM x USW 5279-4	1	6
IT231864	JPN	Solanum tuberosum subsp. tuberosum	Star Ruby	Cultivar	HOKKAI 77 x 87028-6	2	8
IT231866	JPN	Solanum tuberosum subsp. tuberosum	Sinsia	Cultivar		1	7
IT231867	JPN	Solanum tuberosum subsp. tuberosum	Tokachikogane	Cultivar	R392-3 x 69095-17	2	8
IT231872	JPN	Solanum tuberosum subsp. tuberosum	Self-Kid Purple	Cultivar		1	2
IT231873	JPN	Solanum tuberosum subsp. tuberosum	Self Kid Red	Cultivar		2	2

IT231874	JPN	Solanum tuberosum subsp. tuberosum	Hanashibetsu	Cultivar	W553-4 (adg) x R392-50	1	6
IT231876	JPN	Solanum tuberosum subsp. tuberosum	Destroyah	Cultivar		1	3
IT231877	JPN	Solanum tuberosum subsp. tuberosum	Dorothy	Cultivar		1	6
IT231879	BGR	Solanum tuberosum subsp. tuberosum	K144650	Breeding line		1	3
IT231880	BGR	Solanum tuberosum subsp. tuberosum	K144651	Breeding line		2	8
IT231881	BGR	Solanum tuberosum subsp. tuberosum	K144652	Breeding line		2	8
IT231882	BGR	Solanum tuberosum subsp. tuberosum	K144653	Breeding line		2	8
IT231883	BGR	Solanum tuberosum subsp. tuberosum	K144654	Breeding line		2	8
IT231884	BGR	Solanum tuberosum subsp. tuberosum	K144655	Breeding line		2	8
IT231885	BGR	Solanum tuberosum subsp. tuberosum	K144656	Breeding line		1	8
IT231886	BGR	Solanum tuberosum subsp. tuberosum	K144657	Breeding line		2	8
IT231887	BGR	Solanum tuberosum subsp. tuberosum	K144660	Breeding line		2	7
IT231888	BGR	Solanum tuberosum subsp. tuberosum	K144661	Breeding line		1	7
IT231889	BGR	Solanum tuberosum subsp. tuberosum	K144662	Breeding line		2	8
IT231890	BGR	Solanum tuberosum subsp. tuberosum	K144663	Breeding line		1	2
IT231891	BGR	Solanum tuberosum subsp. tuberosum	K144672	Breeding line		2	8
IT231892	JPN	Solanum tuberosum subsp. tuberosum	Shadow queen	Cultivar	KITAMURASAKI open pollinated	1	3
IT231894	USA	Solanum tuberosum subsp. tuberosum	Irish Cobbler	Cultivar	EARLY ROSE mutant	1	7
IT231895	USA	Solanum tuberosum subsp. tuberosum	Atlantic	Cultivar	WAUSEON x LENAPE	1	3
IT231896	JPN	Solanum tuberosum subsp. tuberosum	Dejima	Cultivar	HOKKAI 31 x UNZEN	1	1
IT231898	USA	Solanum tuberosum subsp. tuberosum	Superior	Cultivar	USDA 96-56 x MINN. 59-44	1	6
IT231899	KOR	Solanum tuberosum subsp. tuberosum	Jopung	Cultivar	Resy* Superior	1	6
IT231900	KOR	Solanum tuberosum subsp. tuberosum	Jishim	Cultivar	Daekwan48 * B6603-6	1	3
IT231901	KOR	Solanum tuberosum subsp. tuberosum	Chubaek	Cultivar	H83011-3 * Superior	1	6
IT231902	KOR	Solanum tuberosum subsp. tuberosum	Gawon	Cultivar	Namsuh * Konahubuki	1	7
IT231903	KOR	Solanum tuberosum subsp. tuberosum	Jaseo	Cultivar	Daekwan48 * B6603-6	1	3
IT231904	KOR	Solanum tuberosum subsp. tuberosum	Jowon	Cultivar	AG54071 * Katahdin	1	6
IT231905	KOR	Solanum tuberosum subsp. tuberosum	Gahwang	Cultivar	Atlantic * AG54071	1	3
IT231906	KOR	Solanum tuberosum subsp. tuberosum	Chugang	Cultivar	H83005-2 * Superior	1	1
IT231907	KOR	Solanum tuberosum subsp. tuberosum	Chuyoung	Cultivar	Dejima * HRB-31	1	1
IT231908	KOR	Solanum tuberosum subsp. tuberosum	Haryung	Cultivar	Atlantic* Superior	1	1
IT231909	KOR	Solanum tuberosum subsp. tuberosum	Seohong	Cultivar	Jashim*93K61-5	1	3
IT231910	KOR	Solanum tuberosum subsp. tuberosum	Goun	Cultivar	Lemhi russet * Chubaek	1	1
IT231912	KOR	Solanum tuberosum subsp. tuberosum	Sinnamjak	Cultivar	Irish cobbler * Katahdin	1	6
IT231924	KOR	Solanum tuberosum subsp. tuberosum	HAt-22	Breeding line		1	6

IT231925	USA	Solanum tuberosum subsp. tuberosum	1015095-10	Breeding line		2	8
IT231926	USA	Solanum tuberosum subsp. tuberosum	1015095-4	Breeding line		1	7
IT231940	USA	Solanum tuberosum subsp. tuberosum	44-1016-10	Breeding line		1	1
IT231944	USA	Solanum tuberosum subsp. tuberosum	8TB-8	Breeding line		2	6
IT231947	USA	Solanum tuberosum subsp. tuberosum	9-3	Breeding line		1	3
IT231952	USA	Solanum tuberosum subsp. tuberosum	AL46-9	Breeding line		1	3
IT231957	USA	Solanum tuberosum subsp. tuberosum	B5089-17	Breeding line		1	6
IT231958	USA	Solanum tuberosum subsp. tuberosum	B5306-40	Breeding line		1	6
IT231959	USA	Solanum tuberosum subsp. tuberosum	B6518-4	Breeding line		1	7
IT231960	USA	Solanum tuberosum subsp. tuberosum	B6527-33	Breeding line		1	7
IT231962	USA	Solanum tuberosum subsp. tuberosum	USDA B 6603-6	Breeding line	USDA B 04063 x LENAPE	1	3
IT231963	USA	Solanum tuberosum subsp. tuberosum	OCEANIA	Breeding line	DT 5997-1R x USDA B 5283-5	1	3
IT231964	USA	Solanum tuberosum subsp. tuberosum	B6987-4	Breeding line		1	3
IT231965	USA	Solanum tuberosum subsp. tuberosum	B8148-1	Breeding line		1	7
IT231971	USA	Solanum tuberosum subsp. tuberosum	B9361-4	Breeding line		1	3
IT231977	USA	Solanum tuberosum subsp. tuberosum	B9471-2	Breeding line		1	6
IT231979	USA	Solanum tuberosum subsp. tuberosum	B9474-2	Breeding line		1	3
IT231980	USA	Solanum tuberosum subsp. tuberosum	B9481-3	Breeding line		1	3
IT231981	USA	Solanum tuberosum subsp. tuberosum	B9482-3	Breeding line		1	6
IT231982	USA	Solanum tuberosum subsp. tuberosum	B9483-2	Breeding line		1	6
IT231983	USA	Solanum tuberosum subsp. tuberosum	BR6315-4	Breeding line		1	6
IT231984	JPN	Solanum tuberosum subsp. tuberosum	Hokkai 79	Breeding line		1	6
IT243072	USA	Solanum tuberosum subsp. tuberosum	DTO-50	Breeding line		1	1
IT243076	USA	Solanum tuberosum subsp. tuberosum	NORWIS	Breeding line	RD 289-18 x MONONA	1	6
IT243084	JPN	Solanum tuberosum subsp. tuberosum	Unknown	Cultivar		2	8
IT243085	JPN	Solanum tuberosum subsp. tuberosum	Unknown	Cultivar		2	8
IT243087	USA	Solanum tuberosum subsp. tuberosum	ND2008-2	Breeding line		1	6
IT243089	USA	Solanum tuberosum subsp. tuberosum	ND651-9	Breeding line		1	6
IT243090	USA	Solanum tuberosum subsp. tuberosum	ND860-2	Breeding line	ND78-3 * ND9583-1	1	3
IT243091	JPN	Solanum tuberosum subsp. tuberosum	NORIN NO.1	Cultivar	IRISH COBBLER x DEODARA	1	3
IT243092	JPN	Solanum tuberosum subsp. tuberosum	Norin No.2	Cultivar	IRISH COBBLER x PEPO	2	8
IT243096	USA	Solanum tuberosum subsp. tuberosum	PAS3064-13	Breeding line		2	8
IT243097	USA	Solanum tuberosum subsp. tuberosum	Penn 71	Breeding line	PENNCHIP x KENNEBEC	1	6
IT243123	USA	Solanum tuberosum subsp. tuberosum	T44-2	Breeding line		1	6
IT243124	USA	Solanum tuberosum subsp. tuberosum	T44-3	Breeding line		1	3

IT243126	USA	Solanum tuberosum subsp. tuberosum	T44-6	Breeding line		1	2
IT243130	USA	Solanum tuberosum subsp. tuberosum	U255-2	Breeding line		1	3
IT243132	USA	Solanum tuberosum subsp. tuberosum	W718	Breeding line		1	6
IT243134	USA	Solanum tuberosum subsp. tuberosum	W726	Breeding line		1	2
IT243137	JPN	Solanum tuberosum subsp. tuberosum	WB902241-1	Breeding line		1	2
IT243138	USA	Solanum tuberosum subsp. tuberosum	WHS17	Breeding line		2	8
IT243139	USA	Solanum tuberosum subsp. tuberosum	B5031-18	Breeding line		1	7
IT243141	KOR	Solanum tuberosum subsp. tuberosum	K2-48	Breeding line		2	8
IT243142	USA	Solanum tuberosum subsp. tuberosum	S1-8	Breeding line		1	6
IT243145	JPN	Solanum tuberosum subsp. tuberosum	Unknown	Cultivar		1	6
IT243196	CHN	Solanum tuberosum subsp. tuberosum	Kuksin 4	Cultivar		1	7
IT243197	CHN	Solanum tuberosum subsp. tuberosum	Kuksin 14	Cultivar		2	8
IT243201	USA	Solanum tuberosum subsp. tuberosum	NORVALLEY	Breeding line	NORCHIP x ND 860-2	1	3
IT243206	CHN	Solanum tuberosum subsp. tuberosum	Nea 303	Cultivar		1	6
IT243208	CHN	Solanum tuberosum subsp. tuberosum	Kuksin2	Cultivar		1	8
IT243218	KOR	Solanum tuberosum subsp. tuberosum	Daekwan 31	Breeding line		1	7
IT243219	KOR	Solanum tuberosum subsp. tuberosum	Daekwan 39	Breeding line	Irish cobbler * Tawa	2	8
IT243220	KOR	Solanum tuberosum subsp. tuberosum	713101	Breeding line	Irish cobbler * 257-02	2	8
IT243227	KOR	Solanum tuberosum subsp. tuberosum	75A15-530	Breeding line	Daekwan31 * Merrimack	1	2
IT243230	KOR	Solanum tuberosum subsp. tuberosum	75A15-597	Breeding line	Daekwan31 * Merrimack	1	7
IT243232	KOR	Solanum tuberosum subsp. tuberosum	75A16-670	Breeding line	Daekwan31 * Katahdin	1	7
IT243233	KOR	Solanum tuberosum subsp. tuberosum	75A16-690	Breeding line	Daekwan31 * Katahdin	1	7
IT243235	KOR	Solanum tuberosum subsp. tuberosum	75A33-1029	Breeding line	Simabara * Merrimack	1	7
IT243236	KOR	Solanum tuberosum subsp. tuberosum	75A57-63	Breeding line	Saco * Tachibana	1	3
IT243240	KOR	Solanum tuberosum subsp. tuberosum	75B15-389	Breeding line	Cherokee * Wheeler	1	6
IT243249	KOR	Solanum tuberosum subsp. tuberosum	76B54-2	Breeding line	Cherokee * Platle	1	6
IT243258	KOR	Solanum tuberosum subsp. tuberosum	78E54-10	Breeding line	CIP575015 * Superior	1	7
IT243260	KOR	Solanum tuberosum subsp. tuberosum	78E54-3	Breeding line	CIP575015 * Superior	1	2
IT243261	KOR	Solanum tuberosum subsp. tuberosum	78E54-5	Breeding line	CIP575015 * Superior	1	6
IT243262	KOR	Solanum tuberosum subsp. tuberosum	Daekwan 50	Breeding line	CIP575015 * Superior	1	2
IT243264	KOR	Solanum tuberosum subsp. tuberosum	Daekwan 48	Breeding line	R521-7 * Alamo	1	3
IT243268	KOR	Solanum tuberosum subsp. tuberosum	83H58-4	Breeding line	78E54-1 * Tachibana	1	6
IT243269	KOR	Solanum tuberosum subsp. tuberosum	Daekwan 53	Breeding line	Snowchip * Katahdin	1	3
IT243270	KOR	Solanum tuberosum subsp. tuberosum	83I14-6	Breeding line	S9656 * Arkula	1	2
IT243272	KOR	Solanum tuberosum subsp. tuberosum	86J43-1	Breeding line	Denali * Wheeler	1	7

IT243273	KOR	Solanum tuberosum subsp. tuberosum	86J79-2	Breeding line	Denali * Benimaru	1	3
IT243276	KOR	Solanum tuberosum subsp. tuberosum	Daekwan 55	Breeding line	Lemhi russet * Doyoshiro	1	3
IT243278	KOR	Solanum tuberosum subsp. tuberosum	86J96-4	Breeding line	Dejima * Alamo	1	2
IT243279	KOR	Solanum tuberosum subsp. tuberosum	86J97-4	Breeding line	Dejima * Doyoshiro	1	2
IT243280	KOR	Solanum tuberosum subsp. tuberosum	Daekwan 60	Breeding line	CIP575015 * Katahdin	1	7
IT243281	KOR	Solanum tuberosum subsp. tuberosum	Daekwan 59	Breeding line	CIP575015 * Katahdin	1	1
IT243282	KOR	Solanum tuberosum subsp. tuberosum	Daekwan 58	Breeding line	CIP575015 * CIP3780172	1	3
IT243284	KOR	Solanum tuberosum subsp. tuberosum	88J00-4	Breeding line	Superior * Katahdin	1	6
IT243285	KOR	Solanum tuberosum subsp. tuberosum	88K60-4	Breeding line	CIP575015 * Misang	1	6
IT243287	KOR	Solanum tuberosum subsp. tuberosum	88K63-3	Breeding line		1	6
IT243288	KOR	Solanum tuberosum subsp. tuberosum	88K71-4	Breeding line	Misang * Katahdin	1	6
IT243289	KOR	Solanum tuberosum subsp. tuberosum	88K77-1	Breeding line	Kumsisuh * Saco	1	6
IT243291	KOR	Solanum tuberosum subsp. tuberosum	89L12-2	Breeding line	Daekwan48 * Nook sack	1	6
IT243293	KOR	Solanum tuberosum subsp. tuberosum	Daekwan 65	Breeding line	Daekwan48 * Konahubuki	1	2
IT243294	KOR	Solanum tuberosum subsp. tuberosum	Daekwan 62	Breeding line	Konahubuki * 83H58-4	1	7
IT243313	KOR	Solanum tuberosum subsp. tuberosum	H87511-4	Breeding line		1	7
IT243316	KOR	Solanum tuberosum subsp. tuberosum	Gosi1	Breeding line	H83520-3 * Superior	1	1
IT243317	KOR	Solanum tuberosum subsp. tuberosum	Gosi5	Breeding line	H83520-3 * Superior	1	1
IT243333	KOR	Solanum tuberosum subsp. tuberosum	JI-11	Breeding line		1	6
IT243410	KOR	Solanum tuberosum subsp. tuberosum	C-20	Landrace		1	3
IT243411	KOR	Solanum tuberosum subsp. tuberosum	C-27	Landrace		1	1
IT243419	KOR	Solanum tuberosum subsp. tuberosum	Janggijeok	Cultivar		1	3
IT243424	KOR	Solanum tuberosum subsp. tuberosum	265	Landrace		1	3
IT243428	KOR	Solanum tuberosum subsp. tuberosum	98JaelaeA	Landrace		1	1
IT243429	KOR	Solanum tuberosum subsp. tuberosum	Ulleung Native	Landrace		1	1
IT243430	KOR	Solanum tuberosum subsp. tuberosum	98JaelaeB	Landrace		1	1
IT243431	KOR	Solanum tuberosum subsp. tuberosum	98JaelaeC	Landrace		1	2
IT243432	KOR	Solanum tuberosum subsp. tuberosum	Hongcheon Jaelae A	Landrace		1	6
IT243433	KOR	Solanum tuberosum subsp. tuberosum	Hongcheon Jaelae B	Landrace		1	3
IT243434	KOR	Solanum tuberosum subsp. tuberosum	Hongcheon Jaelae C	Landrace		1	1
IT243435	CHN	Solanum tuberosum subsp. tuberosum	Matdou	Cultivar		1	1
IT243436	CHN	Solanum tuberosum subsp. tuberosum	Y	Cultivar		1	6
IT243440	USA	Solanum tuberosum subsp. tuberosum	Unknown	Breeding line		1	5
IT243441	USA	Solanum tuberosum subsp. tuberosum	Unknown	Breeding line		1	3
IT243442	USA	Solanum tuberosum subsp. tuberosum	Unknown	Breeding line		1	3

IT243445	NLD	Solanum tuberosum subsp. tuberosum	SARA58-K1	Breeding line		1	5
IT243447	NLD	Solanum tuberosum subsp. tuberosum	SARA152-D4	Breeding line		1	5
IT243448	NLD	Solanum tuberosum subsp. tuberosum	SH83-92-488	Breeding line		1	5
IT243449	NLD	Solanum tuberosum subsp. tuberosum	RH89-039-16	Breeding line		1	7
IT243450	KOR	Solanum tuberosum subsp. tuberosum	Daekwan1-73	Breeding line		1	3
IT243452	KOR	Solanum tuberosum subsp. tuberosum	Daekwan1-75	Breeding line		1	3
IT243453	KOR	Solanum tuberosum subsp. tuberosum	Daekwan1-76	Breeding line		1	1
IT243454	KOR	Solanum tuberosum subsp. tuberosum	Daekwan1-77	Breeding line	Daekwan62*Daekwan55	1	6
IT243455	KOR	Solanum tuberosum subsp. tuberosum	Daekwan1-79	Breeding line	Atlantic*Daekwan48	1	3
IT244310	KOR	Solanum tuberosum subsp. tuberosum	Daekwan2-14	Breeding line	Atlantic*Dejima	1	6
IT244311	KOR	Solanum tuberosum subsp. tuberosum	Daekwan2-15	Breeding line	Dejima*Alpha	1	1
IT244312	KOR	Solanum tuberosum subsp. tuberosum	Daekwan1-83	Breeding line	Denali*91M89-3	1	8
IT244316	KOR	Solanum tuberosum subsp. tuberosum	Daekwan1-87	Breeding line	Daekwan62*93N87-7	1	7
IT244317	KOR	Solanum tuberosum subsp. tuberosum	Daekwan2-16	Breeding line	Shinnamjak*Daekwan2-3	1	1
IT244318	KOR	Solanum tuberosum subsp. tuberosum	Daekwan2-17	Breeding line	Atlantic*Daekwan2-3	1	1
IT244968	KOR	Solanum tuberosum subsp. tuberosum	Haba	Breeding line		1	3
IT244970	KOR	Solanum tuberosum subsp. tuberosum	Daekwan1-99	Breeding line		1	1
IT244975	KOR	Solanum tuberosum subsp. tuberosum	Daekwan2-12	Breeding line		1	1
IT245120	BGR	Solanum tuberosum subsp. tuberosum	Agriya	Cultivar	Quarta * Semlo	1	6
IT245121	BGR	Solanum tuberosum subsp. tuberosum	K133550	Breeding line		1	2
IT245122	BGR	Solanum tuberosum subsp. tuberosum	K133551	Breeding line		1	2
IT245123	BGR	Solanum tuberosum subsp. tuberosum	K133552	Breeding line		2	8
IT245124	BGR	Solanum tuberosum subsp. tuberosum	K133554	Breeding line		2	8
IT245125	JPN	Solanum tuberosum subsp. tuberosum	Kita Murasaki	Cultivar	Shimakei571 * Shimakei561	1	3
IT245202	KOR	Solanum tuberosum subsp. tuberosum	Daekwan1-89	Breeding line		1	3
IT245203	KOR	Solanum tuberosum subsp. tuberosum	Daekwan1-90	Breeding line		1	8
IT245205	KOR	Solanum tuberosum subsp. tuberosum	Daekwan1-92	Breeding line		1	7
IT245207	KOR	Solanum tuberosum subsp. tuberosum	Daekwan1-94	Breeding line		1	3
IT245209	KOR	Solanum tuberosum subsp. tuberosum	Daekwan1-96	Breeding line		1	3
IT245211	KOR	Solanum tuberosum subsp. tuberosum	C-10	Landrace		1	3
IT245212	KOR	Solanum tuberosum subsp. tuberosum	C-12	Landrace		1	1
IT245213	KOR	Solanum tuberosum subsp. tuberosum	C-13	Landrace		1	3
IT245216	KOR	Solanum tuberosum	C-23	Landrace		1	3
IT245217	KOR	Solanum tuberosum	C-25	Landrace		1	3
IT245218	KOR	Solanum tuberosum subsp. tuberosum	C-28	Landrace		1	1

IT245219	KOR	Solanum tuberosum subsp. tuberosum	C-29	Landrace		1	3
IT245221	KOR	Solanum tuberosum subsp. tuberosum	C-9	Landrace		1	7
IT245222	KOR	Solanum tuberosum subsp. tuberosum	C-5	Landrace		1	3
IT245223	KOR	Solanum tuberosum subsp. tuberosum	C-4	Landrace		1	3
IT245224	KOR	Solanum tuberosum subsp. tuberosum	C-8	Landrace		1	3
IT245226	KOR	Solanum tuberosum subsp. tuberosum	C-17	Landrace		1	3
IT245915	JPN	Solanum tuberosum subsp. tuberosum	Koganemaru	Cultivar	Musamaru*Tokachi Kogane	1	1
IT245920	BGR	Solanum tuberosum subsp. tuberosum	K154673	Breeding line		1	2
IT245921	BGR	Solanum tuberosum subsp. tuberosum	K154674	Breeding line		2	8
IT245922	BGR	Solanum tuberosum subsp. tuberosum	K154675	Breeding line		1	3
IT245923	BGR	Solanum tuberosum subsp. tuberosum	K154676	Breeding line		1	3
IT245924	BGR	Solanum tuberosum subsp. tuberosum	K154677	Breeding line		2	8
IT245925	BGR	Solanum tuberosum subsp. tuberosum	K154678	Breeding line		2	2
IT245926	BGR	Solanum tuberosum subsp. tuberosum	K154679	Breeding line		1	2
IT245927	BGR	Solanum tuberosum subsp. tuberosum	K154680	Breeding line		1	3
IT245929	BGR	Solanum tuberosum subsp. tuberosum	K154682	Breeding line		1	7
IT245930	BGR	Solanum tuberosum subsp. tuberosum	K154683	Breeding line		1	3
IT245931	BGR	Solanum tuberosum subsp. tuberosum	K154684	Breeding line		1	3
IT245932	PER	Solanum tuberosum subsp. tuberosum	Wamantanga	Cultivar		2	5
IT245933	PER	Solanum tuberosum subsp. tuberosum	Mi-Peru	Cultivar	CHATA NEGRA DE HUASAHUASI x GRATA	2	8
IT245934	PER	Solanum tuberosum subsp. tuberosum	Andina	Cultivar	(Grata*Yana Imilla)*Chata Blanca de Huasahusai	2	8
IT245935	PER	Solanum tuberosum subsp. tuberosum	Coktel	Cultivar		2	5
IT245936	PER	Solanum tuberosum subsp. tuberosum	Amarillia Wamantanga	Cultivar		2	5
IT245938	PER	Solanum tuberosum subsp. tuberosum	Tomada	Cultivar		2	8
IT245941	KOR	Solanum tuberosum subsp. tuberosum	Daekwan1-101	Breeding line	DH09-231*unknown	1	3
IT245942	KOR	Solanum tuberosum subsp. tuberosum	Hongyoung	Breeding line	Atlantic*AG34314	1	6
IT245943	KOR	Solanum tuberosum subsp. tuberosum	Daekwan1-103	Breeding line	Atlantic*AG34314	1	3
IT245944	KOR	Solanum tuberosum subsp. tuberosum	Jayoung	Breeding line	Atlantic*AG34314	1	3
IT245946	KOR	Solanum tuberosum subsp. tuberosum	Daekwan1-106	Breeding line	ND651-9*Gemchip	1	7
IT245947	KOR	Solanum tuberosum subsp. tuberosum	Daekwan1-107	Breeding line	Haryung*Daekwan1-73	1	2
IT245948	KOR	Solanum tuberosum subsp. tuberosum	Daekwan1-108	Breeding line	Haryung*Daekwan1-76	1	2
IT245949	KOR	Solanum tuberosum subsp. tuberosum	Daekwan1-109	Breeding line	Rady Ross*Daekwan1-77	1	2
IT245951	KOR	Solanum tuberosum subsp. tuberosum	Daekwan1-111	Breeding line	Daekwan1-83*AVRDC No.40	1	2
IT245955	KOR	Solanum tuberosum subsp. tuberosum	Daekwan2-24	Breeding line	Atlantic*Chudong	1	3
IT245956	KOR	Solanum tuberosum subsp. tuberosum	Bangul	Cultivar	Dejima*Superior	1	2

IT245960	KOR	Solanum tuberosum subsp. tuberosum	Daekwan2-29	Breeding line	Daekwan2-8*Atlantic	1	1
IT245967	PER	Solanum tuberosum subsp. tuberosum	388615.22	Breeding line	B-71-240.2*386614.16	1	3
IT245970	PER	Solanum tuberosum subsp. tuberosum	391002.6	Breeding line	386209.1*386206.4	admixed	8
IT245971	PER	Solanum tuberosum subsp. tuberosum	392633.54	Breeding line	387132.2*387334.5	2	8
IT245972	PER	Solanum tuberosum subsp. tuberosum	392745.7	Breeding line	88.078*XY-20	2	8
IT245973	PER	Solanum tuberosum subsp. tuberosum	393077.159	Breeding line	387348.20*389746.2	1	2
IT245974	PER	Solanum tuberosum subsp. tuberosum	393079.4	Breeding line	387004.13*390357.4	1	3
IT245975	PER	Solanum tuberosum subsp. tuberosum	393371.157	Breeding line	387170.16*389746.2	1	2
IT245976	PER	Solanum tuberosum subsp. tuberosum	395037.107	Breeding line	391004.4*391679.12	1	7
IT245977	PER	Solanum tuberosum subsp. tuberosum	395109.29	Breeding line	391589.26*393079.4	1	7
IT245978	PER	Solanum tuberosum subsp. tuberosum	395112.9	Breeding line	391686.15*393079.4	1	2
IT245979	PER	Solanum tuberosum subsp. tuberosum	395192.1	Breeding line	388611.22*C92.044	1	7
IT245980	PER	Solanum tuberosum subsp. tuberosum	395434.1	Breeding line	388611.22*N93.067	1	3
IT245981	PER	Solanum tuberosum subsp. tuberosum	396004.263	Breeding line	391002.6*393382.64	admixed	8
IT245982	PER	Solanum tuberosum subsp. tuberosum	396038.107	Breeding line	393077.54*393280.64	1	3
IT245983	PER	Solanum tuberosum subsp. tuberosum	396244.17	Breeding line	391580.30*392633.10	2	8
IT245984	PER	Solanum tuberosum subsp. tuberosum	397073.16	Breeding line	392823.4*392820.1	1	7
IT245985	PER	Solanum tuberosum subsp. tuberosum	397073.7	Breeding line	392823.4*392820.1	2	8
IT245986	PER	Solanum tuberosum subsp. tuberosum	397077.16	Breeding line	392025.7*392820.1	1	3
IT301362	JPN	Solanum tuberosum	Hokkai Kogane	Cultivar		2	8
IT301363	JPN	Solanum tuberosum	Me — kuin	Cultivar	May Queen??-GB	2	2
IT301369	JPN	Solanum tuberosum subsp. tuberosum	Ai Yutaka	Cultivar	DEJIMA x CHOUKEI 108	admixed	8
IT301382	USA	Solanum tuberosum	Gold Rush	Cultivar	LEMHI RUSSET x ND 450-3R	2	6
IT301391	PER	Solanum tuberosum	INIA 5	Breeding line		2	5
IT301393	PER	Solanum tuberosum	INIA 7	Breeding line		2	5
IT301394	PER	Solanum tuberosum	INIA 8	Breeding line		2	5
IT301395	PER	Solanum tuberosum	INIA 9	Breeding line		2	5
IT301396	PER	Solanum tuberosum	INIA 10	Breeding line		2	5
IT301397	PER	Solanum tuberosum	INIA 11	Breeding line		2	5
IT301398	PER	Solanum tuberosum	INIA 12	Breeding line		2	5
IT301399	PER	Solanum tuberosum	P.Pwidtype-1	Breeding line		2	5
IT301400	PER	Solanum tuberosum	P.Pwidtype-2	Breeding line		2	5
IT301401	PER	Solanum tuberosum	P.Pwidtype-3	Breeding line		2	5
IT301402	PER	Solanum tuberosum	P.Pwidtype-4	Breeding line		2	5
IT301403	PER	Solanum tuberosum	P.Pwidtype-5	Breeding line		2	5

IT301404	PER	Solanum tuberosum	P.Pwidtype-6	Breeding line		2	5
IT301405	PER	Solanum tuberosum	P.Pwidtype-7	Breeding line		2	5
IT301408	NLD	Solanum tuberosum	HLP 1552	Cultivar		2	8
IT301409	NLD	Solanum tuberosum	HLP 1553	Cultivar		2	8
IT301410	CHN	Solanum tuberosum	Zao dabai	Cultivar		2	8
IT301411	CHN	Solanum tuberosum	Ke xin 13	Cultivar		2	8
IT301412	CHN	Solanum tuberosum	Ke xin 22	Cultivar		2	8
IT301413	KOR	Solanum tuberosum	HT10-44	Breeding line		2	8
IT301423	NLD	Solanum tuberosum	Limosa	Cultivar	DUKE OF YORK x BERLIKUMER GEELTJE	2	1
IT301424	NLD	Solanum tuberosum	Urgenta	Cultivar	FURORE x KATAHDIN	2	8
IT301425	NLD	Solanum tuberosum	Bevelander	Cultivar	BRAVO x PREFERENT	2	8
IT301426	NLD	Solanum tuberosum	Dore	Cultivar	DUKE OF YORK x BIERMA A 7	admixed	8
IT301427	NLD	Solanum tuberosum	Advira	Cultivar	BONA x MPI 44.335/68	2	8
IT301428	NLD	Solanum tuberosum	Alpha	Cultivar		2	8
IT301433	NLD	Solanum tuberosum	Climax	Cultivar	BINTJE x RECORD	2	7
IT301434	NLD	Solanum tuberosum	Saskia	Cultivar	RODE EERSTELING x HERALD	2	8
IT301435	NLD	Solanum tuberosum	Vokal	Cultivar	PRIMURA x RHEINHORT	2	8
IT301436	NLD	Solanum tuberosum	Libertas	Cultivar	RECORD x VEENHUIZEN 31185	2	8
IT301437	NLD	Solanum tuberosum	Record	Cultivar	TRENCTRIA x ENERGIE	2	8
IT301438	NLD	Solanum tuberosum	Vekaro	Cultivar	PIMPERNEL x KATAHDIN	2	8
IT301440	NLD	Solanum tuberosum	Ukama	Cultivar	MARIJKE x SIRTEMA	2	8
IT301443	NLD	Solanum tuberosum	Sante	Cultivar	Y 66-13-636 x AM 66-42	2	8
IT301444	JPN	Solanum tuberosum	Ainoaka	Cultivar	Dejima * Norland	2	8
IT301446	CHN	Solanum tuberosum	Ke xin 1	Cultivar		2	8
IT301447	CHN	Solanum tuberosum	Zhong lian hong	Cultivar		2	8
IT301448	CHN	Solanum tuberosum	Zhongshu 3	Cultivar	JINGFENG 1 x BF67A	2	8
IT301449	NLD	Solanum tuberosum	Kriel	Cultivar		2	8
IT301450	NLD	Solanum tuberosum	Rode Kriel	Cultivar		2	8
IT301454	USA	Solanum demissum	PI218047-6	Wild relative		2	4
IT301458	KOR	Solanum tuberosum	Minseo	Cultivar	B1 x Dejima	2	1
IT301464	KOR	Solanum tuberosum	LB15-04	Breeding line		2	4
IT301465	KOR	Solanum tuberosum	BW15-01	Breeding line		2	4
IT301469	KOR	Solanum tuberosum	BW15-05	Breeding line		2	4
IT301470	KOR	Solanum tuberosum	BW15-06	Breeding line		2	4
IT301471	KOR	Solanum tuberosum	BW15-07	Breeding line		2	4

IT301472	KOR	Solanum tuberosum	BW15-08	Breeding line		2	4
IT301473	KOR	Solanum tuberosum	BW15-09	Breeding line		2	4
IT301474	KOR	Solanum tuberosum	BW15-10	Breeding line		2	4
IT301475	CHN	Solanum tuberosum	Cheongseo 9	Cultivar		2	8
IT301479	KOR	Solanum tuberosum	Daekwan2-41	Breeding line		2	8
IT301483	USA	Solanum acaule subsp. acaule	SA4	Wild relative		2	4
IT301484	USA	Solanum berthaultii	SB1-1	Wild relative		2	4
IT301485	USA	Solanum brevicaule	SB2-1	Wild relative		2	4
IT301486	USA	Solanum cardiophyllum	SC1-1	Wild relative		2	5
IT301487	USA	Solanum chacoense	SC3-12	Wild relative		2	4
IT301488	USA	Solanum hjertingii	SH1-8	Wild relative		2	4
IT301489	USA	Solanum hougasii	SH2-6	Wild relative		2	4
IT301490	USA	Solanum iopetalum	SI1	Wild relative		2	4
IT301491	USA	Solanum jamesii	SJ1	Wild relative		2	5
IT301492	USA	Solanum kurtzianum	SK3	Wild relative		2	4
IT301493	USA	Solanum mochiquense	SM1-3	Wild relative		2	4
IT301494	USA	Solanum microdontum	SM2-1	Wild relative		2	4
IT301495	USA	Solanum pinnatisectum	SP3	Wild relative		2	5
IT301496	USA	Solanum raphanifolium	SR1	Wild relative		admixed	5
IT301497	USA	Solanum stoloniferum	SS1	Wild relative		2	4
IT301498	USA	Solanum verrucosum	SV1-1	Wild relative		2	4
IT301499	USA	Solanum vernei	SV2-3	Wild relative		2	4
HLP0702	KOR	Solanum tuberosum subsp. tuberosum	Sinnamjak	Cultivar	Irish cobbler * Katahdin	2	8
HLP1450	KOR	Solanum tuberosum subsp. tuberosum	New peak	Breeding line	Daekwan2-8*DH09-231	2	8
HLP1484	JPN	Solanum tuberosum subsp. tuberosum	Kitaakari	Cultivar	IRISH COBBLER x TUNIKA	2	8
HLP1498	PER	Solanum tuberosum	PI 210035	Wild relative		2	3
HLP1518	USA	Solanum tuberosum	Atlantic	Cultivar		2	8
HLP1591	NLD	Solanum tuberosum	Vivaks	Cultivar	SIRTEMA x MPI 19268	2	3
HLP1593	NLD	Solanum tuberosum	Marijke	Cultivar	SVP M 194-10 x MPI 19268	2	8

Table S2. List of 24 SSR markers used in this study

Name	motif	F	R	Chromosome location	Annealing Temp. (℃)	Product Size (bp)	PIC
STG0001	(CT)n	CAGCCAACATTTGTACCCCT	ACCCCCACTTGCCATATTTT	XI	61	114 - 142	0.758
STM0019a,b	(AT)*n* (GT)*n* (AT)*n* (GT)*n* (GC)*n* (GT)*n*	AATAGGTGTACTGACTCTCAATG	TTGAAGTAAAAGTCCTAGTATGTG	VI	55	109 - 234	0.795
STG0010	(TG)n	CGATCTCTGCTTTGCAGGTA	GTTCATCACTACCGCCGACT	III	61	156 - 172	0.571
STM0031	(AC)5 ... (AC)3 (GCAC) (AC)2 (GCAC)2	CATACGCACGCACGTACAC	TTCAACCTATCATTTTGTGAGTCG	VII	61	110 - 200	0.742
STG0016	(AGA)n	AGCTGCTCAGCATCAAGAGA	ACCACCTCAGGCACTTCATC	I	61	110 - 160	0.754
STM0037	(TC)5 (AC)6 AA (AC)7 (AT)4	AATTTAACTTAGAAGATTAGTCTC	ATTTGGTTGGGTATGATA	XI	55	66 - 96	0.745
STG0025	(AAAC)*n*	TGGAATCCGAATTACGCTCT	AGGTTTTACCACTCGGGCTT	X	61	191 - 205	0.397
STM1052	(AT)14 GT (AT)4 (GT)6	CAATTTCGTTTTTTCATGTGACAC	ATGGCGTAATTTGATTTAATACGTAA	IX	58	208 - 260	0.710
STI0001	(AAT)*n*	CAGCAAAATCAGAACCCGAT	GGATCATCAAATTCACCGCT	IV	61	177 - 197	0.731
STM1053	(TA)4 (ATC)5	TCTCCCCATCTTAATGTTTC	CAACACAGCATSCAGATCATC	III	58	119 - 174	0.158
STI0003	(ACC)n	ACCATCCACCATGTCAATGC	CTCATGGATGGTGTCATTGG	VIII	61	118 - 192	0.779
STM1064	(TA)12..(TG)4 GT (TG)5	GTTCTTTTGGTGGTTTTCCT	TTATTTCTCTGTTGTTGCTG	II	58	182 - 195	0.493
STI0004	(AAG)n	GCTGCTAAACACTCAAGCAGAA	CAACTACAAGATTCCATCCACAG	VI	61	72 - 102	0.666
STM1104	(TCT)n	TGATTCTCTTGCCTACTGTAATCG	CAAAGTGGTGTGAAGCTGTGA	VIII	61	165 - 181	0.547
STI0012	(ATT)n	GAAGCGACTTCCAAAATCAGA	AAAGGGAGGAATAGAAACCAAAA	IV	61	155 - 205	0.705
STM1106	(ATT)13	TCCAGCTGATTGGTTAGGTTG	ATGCGAATCTACTCGTCATGG	X	61	130 - 193	0.529
STI0014	(TGG)n(AGG)n	AGAAACTGAGTTGTGTTTGGGA	TCAACAGTCTCAGAAAACCCTCT	IX	61	113 - 130	0.591
STM5114	(ACC)n	AATGGCTCTCTCTGTATGCT	GCTGTCCCAACTATCTTTGA	II	61	278 - 323	0.725
STI0030	(ATT)n	TTGACCCTCCAACTATAGATTCTTC	TGACAACTTTAAAGCATATGTCAGC	XI	61	83 - 122	0.647
STM5121	(TGT)n	CACCGGAATAAGCGGATCT	TCTTCCCTTCCATTTGTCA	XI	55	283 - 293	0.405
STI0032	(GGA)n	TGGGAAGAATCCTGAAATGG	TGCTCTACCAATTAACGGCA	V	61	105 - 132	0.772
STM5127	(TCT)n	TTCAAGAATAGGCAAAACCA	CTTTTTCTGACTGAGTTGCCTC	I	55	236 - 271	0.727
STI0033	(AGG)n	TGAGGGTTTTCAGAAAGGGA	CATCCTTGCAACAACCTCCT	VI	61	101 - 131	0.720
STPoAc58	(TA)13	TTGATGAAAGGAATGCAGCTTGTG	ACGTTAAAGAAGTGAGAGTACGAC	V	61	199 - 245	0.270

Table S3. Missing rate (%) of 24 SSR markers in this study

SSR marker	Missing rate
STG0001	3.90%
STM0019ab	3.90%
STG0010	1.70%
STM0031	3.30%
STG0016	0.60%
STM0037	1.70%
STG0025	0.40%
STM1052	0.40%
STI0001	0.20%
STM1053	0.40%
STI0003	0.20%
STM1064	0.20%
STI0004	0.40%
STM1104	2.70%
STI0012	1.70%
STM1106	5.00%
STI0014	0.20%
STM5114	-
STI0030	0.80%
STM5121	0.20%
STI0032	0.60%
STM5127	1.00%
STI0033	0.80%
STPoAc58	1.20%
Mean	1.30%
